# Visualizing stress granule dynamics with an RNA guanine quadruplex targeted ruthenium(ii) peptide conjugate†

**DOI:** 10.1039/d5cb00008d

**Published:** 2025-06-19

**Authors:** Rhianne C. Curley, Lorcan Holden, Tia E. Keyes

**Affiliations:** a School of Chemical Sciences, Life Sciences Institute, Dublin City University Dublin 9 Ireland tia.keyes@dcu.ie

## Abstract

Stress granules (SGs) are membraneless ribonucleoprotein assemblies that form in response to cellular stress. They have been linked to cell survival and cancer progression, though many questions remain regarding their structure, function and therapeutic potential. Live-cell fluorescence imaging is key to advancing understanding of SGs, but there are very few small-molecule probes reported that selectively image these organelles. RNA G-quadruplex (rG4) folding is believed to play a role in initiation of SG formation. Thus, to create a probe for SGs, we conjugated a G4 binding domain peptide from RNA helicase associated with AU-rich element (RHAU) to a luminescent [Ru(bpy)_2_(PIC-COOH)]^2+^, Ru-RHAU. Ru-RHAU is designed to target rG4s and thus SGs in live cells. Studies *in cellulo* demonstrate that Ru-RHAU can induce SG formation in a concentration and time dependent manner and immunolabelling confirmed the complex remains associated with rG4s in the SGs. The SG stimulation is attributed to stabilization of rG4 by Ru-RHAU consistent with rG4's role in SG formation. Ru-RHAU shows low cytotoxicity under imaging conditions, facilitating prolonged observation in live cells. Interestingly, under more intense photoirradiation, Ru-RHAU induces phototoxicity through an apoptotic pathway. Ru-RHAU is a versatile tool for probing SG dynamics and function in cellular stress responses and has heretofore unconsidered potential in phototherapeutic applications targeting SGs.

## Abstract 2

A cell permeable Ru(ii) polypyridyl complex conjugated to G4 binding domain peptide from RNA helicase associated with AU-rich element (RHAU) was developed as a tool to visualize stress granule dynamics in live cells.

## Graphical abstract

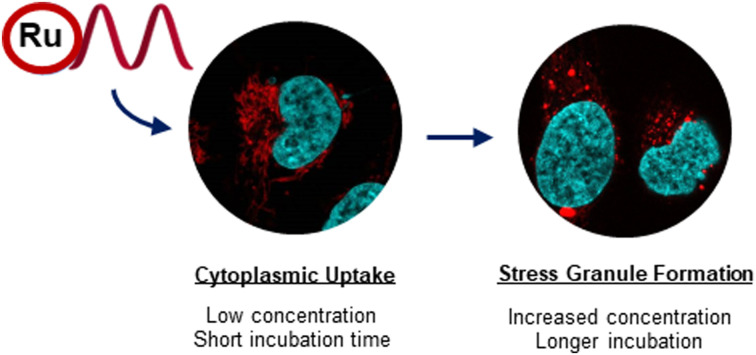

## Introduction

Guanine quadruplexes (G4s) are non-canonical secondary structures that assemble in guanine rich sequences through a combination of Hoogsteen hydrogen bonding, pi stacking and van der Waals forces. They occur in both DNA and RNA sequences. Compared to DNA, guanine in RNA contains a 2′-hydroxyl group at ribose, that facilitates greater intramolecular interaction, increasing RNA G4 (rG4) *in vitro* stability. Another key difference is the rG4 topological preference for parallel structure, attributed to steric constraints from the presence of the 2′ hydroxyl-group.^1^ In recent years, numerous studies have provided evidence for the formation of rG4s in live cell environments including imaging, computational, and molecular biology studies, linking their formation to essential biological processes.^2^ Cytoplasmic rG4s have been implicated in RNA translation, stability, transport, and RNA-binding protein (RBP) sequestering, although their roles and prevalence have yet to be fully elucidated.^3^ In particular, due to RNA's role in translation, rG4s have emerged as an exciting target for small molecule drugs, with a view to stabilize rG4s and inhibit translation by preventing ribosomal activity or translation machinery. Recently, a link has emerged between the formation of cytoplasmic rG4s and stress granules (SGs).^4^ Theoretical and experimental studies indicate that rG4 sequences are ubiquitous in the 5′ and 3′ untranslated regions (UTRs) of mRNAs and that rG4 folding is stimulated in mRNA 3′-UTRs under stress conditions.^5–7^ Furthermore, evidence suggests rG4 folding in UTRs stimulates interactions with RNA-binding proteins seeding the formation of ribonucleoprotein biomolecular condensates including stress granules (SGs). Recent studies have provided direct evidence for rG4 role in the assembly of SGs.^8,9^ For example Turner *et al.* have reported that G3BP1, a key SG nucleating protein, interacts with rG4s in a stress-dependent manner.^10^

SGs are non-membranous, phase-separated organelles that assemble in response to various cell stress response pathways. Some stressors include heat shock or arsenic salt concentrations, and exposure to UV-light. By sequestering UTRs into condensates, SGs are believed to provide a protective effect for sequestered mRNAs. SG formation in response to cellular stress has notable advantages for cell survival by reducing energy expenditure and minimizing damage. However, SG role and function is not yet fully elucidated.

Aberrant, persistent SGs result from SG dysregulation and have been linked to cancer progression as well as several human neurodegenerative disorders, including amyotrophic lateral sclerosis (ALS).^11–13^ While it is clear that SGs are of great importance to cellular homeostasis and survival under stress conditions. There are many open questions regarding their structure, mechanism and dynamics of formation and disassembly. There are currently few probes available for their study in live cells. Immunofluorescent labelling or fluorescence *in situ* hybridization (FISH) are the most widely used approaches but they are not amenable to live cell studies.^14–16^ Ideally to investigate and visualize the composition of SGs, to monitor their assembly and disassembly in a live cell environment and to unravel their connection to rG4s, small molecule, cell permeable probes that recognize rG4s are required.^17^ To date, the first and only small molecule sensor for imaging SGs in live cells was reported by Shao *et al.* who demonstrated a cell permeable benzothiazole cyanine fluorophore that appears to bind both to RNA and SG protein that led to fluorescence turn-on attributed to viscosity sensing in live HeLa cells.^18^

Ruthenium(ii) polypyridyl probes have emerged as promising imaging agents, due to their excellent photostability, large Stoke shifts and long lived ^3^MLCT excited state, rendering them sensitive to their local environment.^19^ They have been implemented with advanced live imaging techniques such as stimulated depletion microscopy (STED) and structured illumination microscopy (SIM).^20,21^ However, a drawback commonly encountered when Ru(ii) polypyridyl complexes are explored for biological applications is either poor membrane permeability or endosomal entrapment, that prevents interaction with desired biomolecules or organelles and limiting their efficacy and potential. To overcome this, our group has previously demonstrated that judicial ligand design and bio-conjugation, particularly implementation of cell penetrating peptides (CPPs) or signal peptides, can promote permeability and facilitate organelle targeting of these complexes.^22–25^ The use of CPPs promotes cell uptake and can offer selective organelle targeting, for example we have reported that Ru(ii) conjugates of nuclear localization signal, NLS and mitochondrial penetrating peptide, MPP can traverse the cell membrane and localize within the nucleus and mitochondria respectively.^26,27^ Similarly, other researchers have explored the functionalization of Ru(ii) complexes with diverse peptides to study DNA interaction, facilitate cellular uptake or expand their therapeutic applications.^28,29^

Ruthenium(ii) complexes are potent G4 probes, offering advantages in stability, specificity, and photophysical properties.^30^ A number of G4-targeted ruthenium complexes have been reported recently including two G4 targeted probes Ru-PDC3 and Ru-TAP-PDC3 studied by our group.^31,32^ Furthermore Yang *et al.* studied the ultrafast excited state dynamics and light-switching of the commonly studied Ru(ii) complex [Ru(phen)_2_(dppz)]^2+^ in G4 DNA.^33^

Recently, peptides capable of selective G4 binding have shown promise in biological applications but they are, to date, relatively underexplored as targeting vectors.^34^ For example, peptides derived from the RNA helicase associated with AU-rich element (RHAU) contain a helical G4 binding domain with selectivity for parallel G4s, offering exciting prospectives for rG4 monitoring and targeted therapies.^35,36^ RHAU helicase derived peptides and their chemically modified counterparts are effective G4 binding ligands. A key advantage of RHAU peptides as vectors is that they can show very high selectivity for G4 structures and even sub-topology specificity that is very difficult to accomplish in small molecule probes. The 53 amino acid long peptide, Rhau53, binds to various parallel G4s but does not bind to other G4s or duplex DNA.^37^ There are some drawbacks of G4 targeted peptides including limited cellular uptake in their linear form and stability issues in a cell environment. Efforts to directly modify the peptide through stapling have resulted in a locked helical form with increased stability but critically, diminished selectivity for G4s.^38^ Additionally, there is a lack of cell-based studies to investigate the function of peptide-based leads for therapeutic applications.^39^

Herein, we present the first example of a metal-based SG probe comprising a Ru(ii)–peptide bioconjugate, Ru-RHAU, that incorporates a peptide derived from the binding domain of the RHAU helicase protein in order to achieve rG4 binding. The bioconjugate was designed to increase peptide stability, rG4 stabilization and live cell visualization using fluorescence microscopy. We demonstrate that the probe can be used to explore rG4 and SG dynamics in a concentration and time dependent manner, inspired by reports of the RHAU helicase sequestering to SGs as a cellular stress response. Furthermore, we report to the best of our knowledge, the first example of an imaging dye capable of generating and imaging SGs in live cells, an important advancement for understanding SG dynamics.

## Results and discussion

### Synthesis and photophysical characterization

**Scheme 1:**
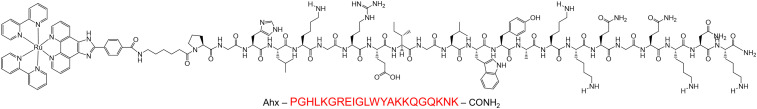
Chemical structure of Ru-RHAU.

**Fig. 1:**
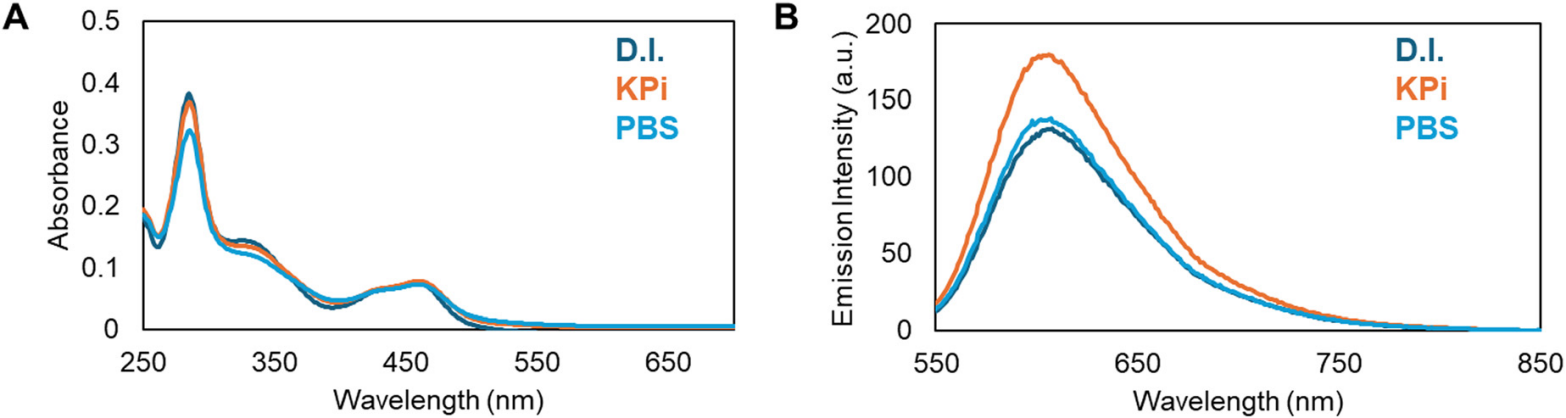
(A) Absorbance and (B) emission spectra of Ru-RHAU (5 μM) in D.I. water (dark blue), KPi buffer (orange) and PBS (light blue), excitation wavelength 490 nm.

The parent complex, [Ru(bpy)_2_(Pic-COOH)]^2+^ (Ru-PIC), also known as Ru-PIP, was prepared and characterized as previously described.^25,40^ The peptide conjugate Ru-RHAU (Scheme 1) was synthesized through NHS coupling in an analogous route to one previously reported by our group,^41^ with structural confirmation from ^1^H NMR, mass spectrometry and HPLC analysis. The peptide, PGHLKGREIGLWYAKKQGQKNK, derived from the N-terminal domain of the RHAU helicase protein was synthesized by Celtek Peptides, incorporating a terminal hexyl amino linker as the site of peptide coupling. Conjugation of the parent complex to the peptide was achieved through a NHS coupling, stirring in a DMF and PBS (pH 7.4) solution. The complex was initially precipitated out as a PF_6_ salt before conversion to chloride salt by stirring overnight on amberlite chloride exchange resin. Successful conjugation and purity of Ru-RHAU was confirmed by HPLC analysis at 280 nm and 450 nm (Fig. S2, ESI†). The structure was confirmed through ^1^H NMR and mass spec analysis (*m*/*z* calculated = 3382.6494, found = 3382.5083) (Fig. S3, ESI†). Photophysical characterization of Ru-RHAU and its conjugate was completed in deionized (D.I.) water, potassium phosphate buffer and MeCN, representative absorbance and luminescence data are shown in Fig. 1. Ru-RHAU shows MLCT absorbance maxima at 460 nm in D.I. water, 455 in KPi buffer (10 mM potassium phosphate and 100 mM KCl) and 458 nm in PBS. When excited at 490 nm, the emission maximum of Ru-RHAU is centred in D.I. at 608 nm, in PBS at 601 nm and in KPi at 600 nm. The emission conforms to mono-exponential decay kinetics of 487 ns in D.I. water, 580 ns in KPi and 584 ns in PBS.

### DNA and RNA binding

**Fig. 2:**
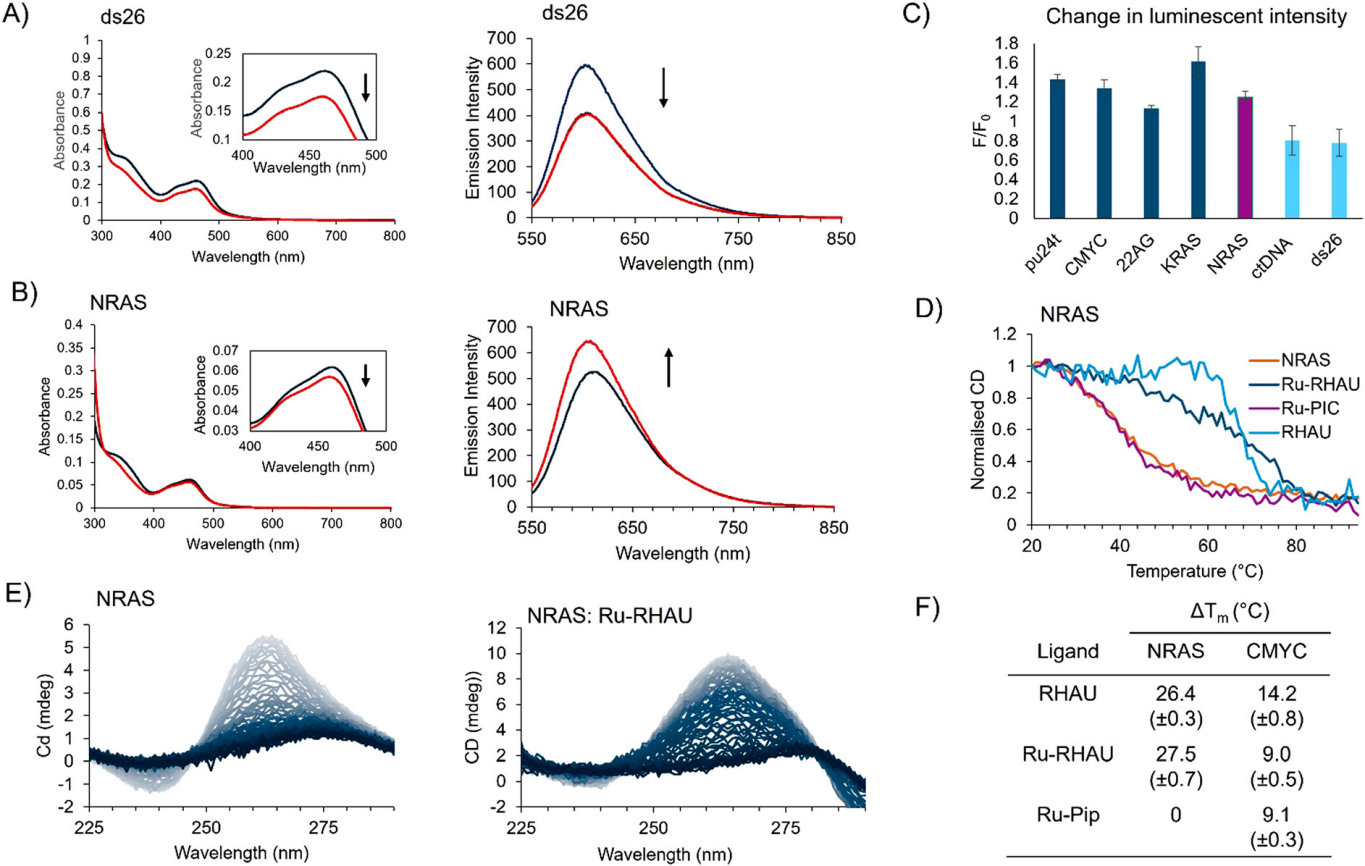
(A) Absorbance and emission titration of Ru-RHAU (10 μM (blue)) and bound to ds26 (30 μM (red)). (B) Absorbance and emission titration of Ru-RHAU (2 μM (blue)) and bound to NRAS (6 μM (red)). (C) Normalized changes in fluorescence upon association with 3 equivalents of oligonucleotides. (D) Normalized changes in the CD signal of NRAS at 265 nm when bound to 2 equivalents of RHAU, Ru-PIC or Ru-RHAU with increasing temperature. (E) Circular dichroism melting curves of NRAS (5 μM) unbound and bound to Ru-RHAU (10 μM) from 20–94 °C. (F) Changes in melting temperatures of NRAS and CMYC when bound to 2 equivalents of RHAU, Ru-PIC or Ru-RHAU.

To compare the DNA and RNA binding capabilities of the Ru-RHAU conjugate, absorbance and luminescence titrations were performed. The oligonucleotides included G4 forming sequences; KRAS, Pu24T, CMYC, 22AG, NRAS and dsDNA (calf thymus DNA (ctDNA) and ds26). The DNA G4 oligonucleotides were annealed in KPi buffer at a DNA strand concentration of 1 mM, prior to use. RNase free buffer was used to prepare stocks of the RNA oligonucleotides with a strand concentration of 0.1 mM, similarly, with the addition of potassium phosphate and potassium chloride. Representative absorption and emission titration plots for Ru-RHAU against dsDNA and NRAS are illustrated in Fig. 2.

Upon addition of DNA oligonucleotides, there are small decreases in the MLCT absorption of Ru-RHAU observed. The absorbance intensity data were fit to a modified Scatchard equation to obtain binding constants, Table 1. Based on control studies, 7 minutes were allowed after each aliquot of nucleic acid before collecting the spectra, to ensure binding equilibrium had been reached. Representative binding curves used to extract binding affinities are shown in Fig. S5 (ESI†). Ru-RHAU showed greatest binding affinity for the parallel quadruplex CMYC with a *K*_b_ of 1.5 (±0.2) × 10^7^. The unmodified 23 amino acid RHAU peptide has been reported to show preferential binding to CMYC (with similar *K*_d_ of 112 nM), this suggests the conjugate is associating, through the peptide, in the same binding mode as the unconjugated peptide.^38^ KRAS and 22AG binding affinities are estimated as 1.0 (± 0.1) × 10^6^ and 5.2 (± 0.8) × 10^6^ respectively. Conjugate binding affinities toward duplex DNA; ctDNA and oligonucleotide ds26 were determined as 6.2 (± 1.2) × 10^5^ and 2.5 (± 0.5) × 10^5^ respectively, *i.e.*, roughly an order of magnitude weaker than for G4 structures. The absorption data for the RNA parallel quadruplex NRAS did not fit well to the Scratchard equation model, (*i.e.* based on residuals and *χ*^2^ values). This is attributed to the uncertainty associated with small absorbance changes observed, which is not surprising given it is the peptide rather than metal center that recognises the NRAS. However, the luminescence response of Ru-RHAU on NRAS binding titration fit well and was used to extract a binding constant *K*_b_ of 1.9 (±0.2) × 10^6^ which is comparable to DNA G4 affinity.

**Table 1:** Binding affinities of Ru-RHAU to a series of oligonucleotides, changes in fluorescence and increases in thermal stability

Sequence^a^	Binding affinities (M^−1^)	SSR^b^ (%)	*F*/*F*_0_	Δ*T*_m_^c^ (°C)
Pu24T	3.2 × 10^5^ (± 0.4)	4.0	1.43 (± 0.5)	17.6 (± 0.6)
CMYC	1.5 × 10^7^ (± 0.2)	6.5	1.34 (± 0.08)	9.0 (± 0.5)
KRAS	1.0 × 10^6^ (± 0.1)	5.3	1.62 (± 0.15)	10.9 (± 1.0)
22AG	5.2 × 10^6^ (± 0.8)	3.7	1.13 (± 0.03)	2.9 (± 0.6)
NRAS	1.9 × 10^6^ (± 0.2)	5.2	1.25 (± 0.06)	26.4 (±0.3)
ctDNA	6.2 × 10^5^ (± 1.2)	4.3	0.80 (± 0.15)	n.d.
ds26	2.5 × 10^5^ (± 0.5)	3.9	0.68 (± 0.14)	n.d.

a Full sequences can be found in Table S1 (ESI).

b SSR = sum of the square residuals, based on deviation from the plots in Fig. S5 (ESI) using the modified Scratchard equation.^42^

c n.d. = not determined.

Luminescence titrations were performed to understand and compare the impact of DNA and G4 association on the photophysical properties of Ru-RHAU. An excitation wavelength of 490 nm was selected as it lies in a pseudo isosbestic point in the absorption data. Interestingly, opposing effects were observed in spectral data on incubation with G4 forming oligos and dsDNA. Hyperchromic responses were observed when Ru-RHAU was bound to the parallel G4s KRAS, Pu24T, and CMYC were 62%, 43% and 34% respectively and 25% for the rG4 NRAS. Whereas a hypochromic effect was observed on dsDNA association of 20% and 32% on saturation binding for ctDNA and ds26 respectively (Fig. 2(C)).

Control experiments on the association of the parent complex, [Ru(bpy)_2_(Pic-COOH)]^2+^ (Ru-PIC), with ctDNA, CMYC and NRAS (Fig. S7 and S8, ESI†) showed that Ru-PIC did not elicit a strong absorbance spectral response on incubation with ctDNA but notably, did result in an increase in luminescent intensity. This contrasts with Ru-RHAU suggesting a different binding mode is at play. It has been reported that Ru(ii) complexes bearing ‘pic-like’ ligands can intercalate between base pairs leading to increase in emission intensities.^43^

Conjugation to the RHAU peptide likely inhibits the pic ligand from intercalating dsDNA in a similar manner to the free complex as the bulky peptide is bound to the pic ligand terminus. A decrease in the Ru-PIC MLCT absorption upon association with CMYC and an increase in luminescent intensity occurred with increasing G4 concentrations. In contrast, the binding of Ru-PIC to NRAS had negligible impact on either the absorbance or emission spectra, indicating weak interaction of the parent complex with this sequence. It is possible that the increased stability of NRAS caused by the 2′ hydroxyl group in ribose may prevent Ru-PIC from binding as effectively when compared to the DNA G4 CMYC. In contrast, the increase in luminescence intensity on Ru-RHAU association with NRAS suggests that conjugate association, *via* the peptide, protects the ruthenium centre, decreasing non-radiative decay rate. This was reflected also in changes in the luminescence lifetime of the metal complex on NRAS binding, the emission decay of the Ru-RHAU in PBS was monoexponential, recorded as 584 ns. Whereas on association with NRAS, (at 1 : 2 ratio of complex to NRAS) the emission decay became biexponential and recorded as 906 ns (23% amplitude) and 422 ns (77%). Changes to the emission intensity and lifetime of Ru-RHAU will be influenced by how the Ru complex is enfolded in the final structure as this may change the rotational/vibrational freedom or exclude oxygen or quenchers from the Ru environment, all influencing its non radiative decay.

### Circular dichroism melting studies

The ability of Ru-RHAU to stabilize G4 DNA and RNA was investigated by studying the melting temperature (*T*_m_) of different topologies on Ru-RHAU binding. KRAS, Pu24T and 22AG oligonucleotides were studied in buffer solution of 10 mM potassium phosphate and 100 mM KCl. Due to the high stability of CMYC and NRAS in buffers with high potassium concentrations, a weaker stabilizing buffer was required to obtain melting temperature curves, comprised of LiCl (10 mM) and lithium cacodylate (100 mM) at pH 7.

In the case of the parallel G4s, Pu24T and KRAS, Ru-RHAU clearly stabilized the G-quadruplex evident in Δ*T*_m_ of 17.6 (± 0.6) °C for Pu24T and 10.9 (± 1.0) °C for KRAS. Ru-RHAU caused Δ*T*_m_ of 9.0 (± 0.5) °C in CMYC. Interestingly, although Ru-RHAU showed high affinity for 22AG, Ru-RHAU binding did not result in notable G4 stabilization where Δ*T*_m_ was only 2.9 (± 0.6) °C. Also of note, the CD spectra of 22AG indicates that on binding to Ru-RHAU, conformational changes from a hybrid structure to an anti-parallel conformation occur. The minimal changes in Δ*T*_m_ observed could be the result of differences in the thermal stability between different conformations. In contrast, Ru-RHAU induces Δ*T*_m_ of 26.4 (± 0.3) °C in NRAS indicating considerable stabilization of the RNA G4s structure.

In controls, Δ*T*_m_ induced by the parent complex and unconjugated RHAU peptide were separately compared for NRAS and CMYC (Fig. 2). Upon incubation with 2 equivalents of RHAU a very strong stabilization of 27.5 (± 0.7) °C was observed for CMYC. The extent of stabilization matches very closely that of the Ru-RHAU conjugate, consistent with the binding constant reported above, indicating similar binding in each case. Conversely, when parent complex, Ru-PIC, was incubated with NRAS no stabilization was observed, and a moderate increase observed for CMYC Δ*T*_m_ of 9.1 (± 0.3) °C. Association of RHAU with CMYC resulted in an increase in Δ*T*_m_ of 14.2 (± 0.8) °C as shown in Fig. 2(F). Thus, we conclude in both cases, that peptide binding is driving stabilization rather than metal complex association.

### Cellular uptake and assembly of stress induced foci by Ru-RHAU

**Fig. 3:**
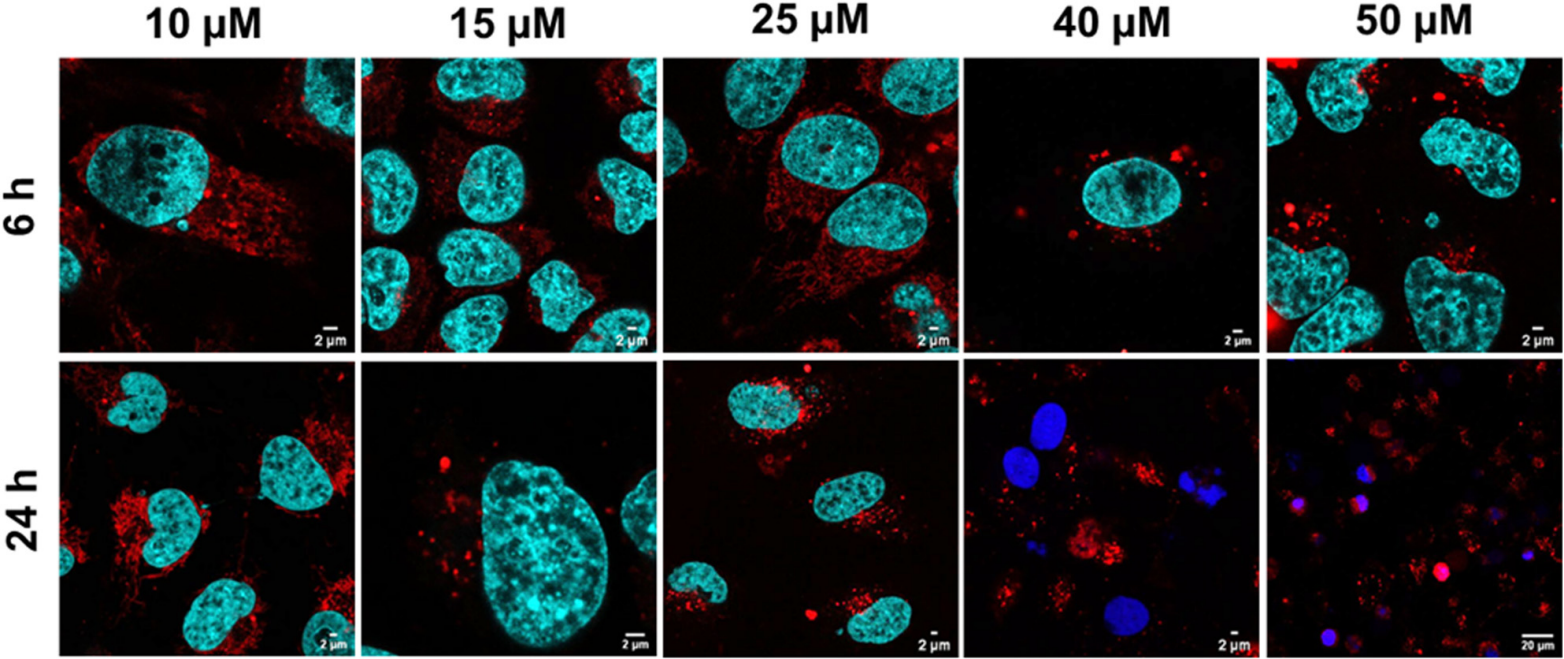
Confocal imaging showing the uptake of Ru-RHAU in live HeLa cells at varying concentrations after 6 and 24 h incubation where DRAQ7 uptake (blue) is evident in cells treated with 40 μM and 50 μM Ru-RHAU for 24 h, confirming cytotoxicity. Hoechst 33342 (cyan) stains the nucleus of healthy cells.

Cellular uptake was assessed by incubating HeLa cells with Ru-RHAU over a range of concentrations and incubation times. Fig. 3 shows the assembly of large foci in the cytoplasm on uptake of Ru-RHAU that is both concentration and time dependent. Notably, large foci (approx. 2 μm) form between 30–40 μM Ru-RHAU after a relatively short incubation of 6 h incubation (Fig. 3 and Fig. S11, ESI†) and at *ca.* 15 μM after a 24 h incubation. In both cases the foci are thought to be assembly of stress granules (SGs). Uptake of DRAQ7 (3 μM) at 40 and 50 μM Ru-RHAU (24 h) indicates cells are dead or damaged under these conditions.

Two time/concentration points were then selected for intracellular imaging of the SGs: 6 h incubation at concentration of 40 μM and a 24 h incubation with a concentration of 25 μM. These concentrations were selected for study since punctate foci could be readily observed without DRAQ7 uptake, indicating the cells remain in good health. SGs have been reported to form independent of the cell cycle phase, and preliminary imaging corroborates this finding (Fig. S14, ESI†). To explore if uptake and SG stimulation is cell line dependent, MCF-7 breast cancer cells were treated with Ru-RHAU at 25 μM (24 h) and 40 μM (6 h) and similar large cytoplasmic foci were observed (Fig. S16, ESI†), confirming induction of SGs by Ru-RHAU and that it is not unique to HeLa cells. It is tempting to speculate that SG induction by Ru-RHAU is attributed to the propensity for Ru-RHAU to stabilize rG4 structure as demonstrated in solution *vide supra*. Since rG4s are purported to provide the seed for SG assembly if these structures are stabilized, they will likely provide a thermodynamic impetus for SG assembly.^4^

Ru-RHAU was found to be readily permeable to live cells, without addition of solvent or other permeant. To understand this, uptake was evaluated after the cells were treated with a combination of metabolic inhibitor oligomycin and 2-deoxy-d-glucose (2DG) to determine if cellular uptake is energy-dependent. When cells are treated with oligomycin, they become more reliant on glycolysis for energy production. 2DG further disrupts this process by inhibiting glycolysis, thus combining both inhibitors creates an energy-starved environment.^44^ Confocal imaging of cells pre-treated with oligomycin and 2DG for 40 minutes before a 24-h incubation with 25 μM Ru-RHAU confirmed strong emission of the complex from the cell interior (Fig. S16, ESI†), indicating uptake occurs independent of oligomycin/2DG treatment suggesting it is an energy-independent, *i.e.* passive process.

### Ru-RHAU localization: from lysosomes to stress granules

Since the RHAU protein can be found in both the nucleus and cytoplasm of cells,^45^ co-localization studies were performed to confirm the exact location of the punctate Ru-RHAU staining we observe in live HeLa cells. Confocal imaging of co-staining with commercial lysosome dye LysoTracker Deep Red confirmed that when Ru-RHAU is not sequestered within SGs, it localizes in the lysosomes of HeLa cells at incubation times of 6 h (40 μM Ru-RHAU, Pearson's coefficient *r* = 0.47 ± 0.06) or 24 h (25 μM Ru-RHAU, Pearson's coefficient *r* = 0.52 ± 0.08) as shown in Fig. S17 and S18 (ESI†). This is not surprising as lysosomes are closely linked to SGs, with lysosomal damage reportedly causing cell stress and SG assembly.^46^ Moreover, SG marker G3BP1 has been shown to co-localize with the LAMP-1 lysosome protein.^47–49^

### Super-resolution imaging shows punctate distribution in stress granules

**Fig. 4:**
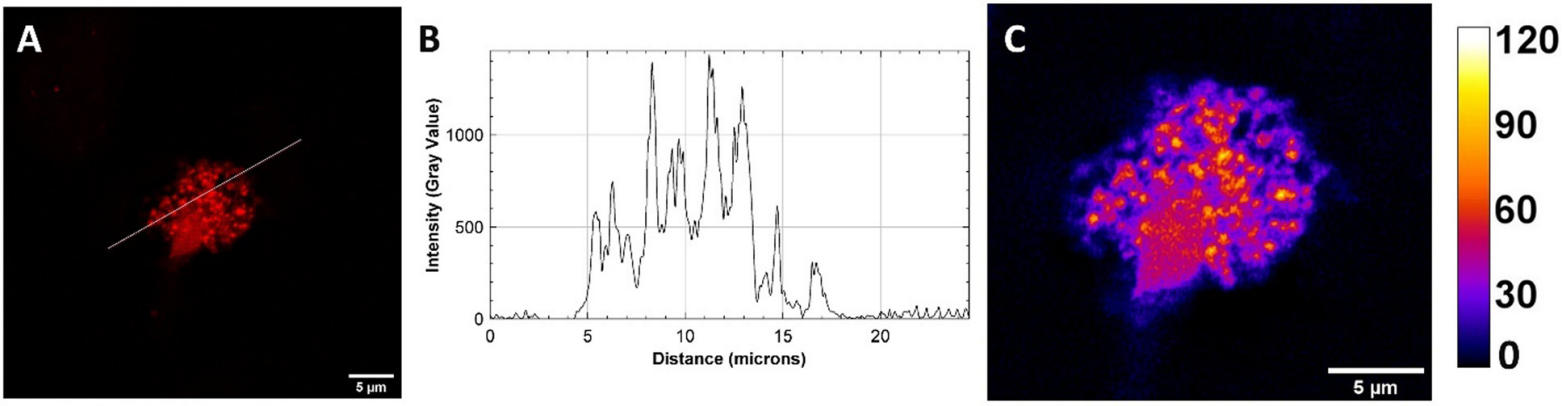
(A) Super-resolution image (Zeiss Airyscan) of a fixed HeLa cell with Ru-RHAU (25 μM, 24 h) where the white line indicates a region of interest used to generate (B) the corresponding plot profile. A zoomed image of the SG with intensity map representing relative gray scale intensity is shown in (C). Scale bars measure 5 μm.

To obtain higher resolution images of the SGs and gain a better insight into the dynamics of the Ru-RHAU induced SGs, airyscanning technology was employed using the ZEISS LSM 980 microscope. This method uses a 32-channel GaAsP area detector instead of the traditional single photomultiplier detector used in conventional confocal microscopy to achieve a lateral resolution of approximately 120 nm, a significant enhancement in comparison to conventional confocal microscopy (250 nm).^50^ As shown in Fig. 4(B), imaging in super-resolution mode allows us to resolve the structure of the stress granules, revealing they are made up of numerous smaller foci. Further analysis of foci in HeLa cells treated with Ru-RHAU at 40 μM (6 h) or 25 μM (24 h) measuring 150–160 foci (per condition) ranging from 0–10 μm^2^ indicate SGs are of similar size across both conditions with average foci of 5.33 ± 0.45 and 5.66 ± 0.39 for 40 μM (6 h) and 25 μM (24 h) respectively. As shown in Fig. 4(C) the concentration of Ru-RHAU varies within the SGs, with distinct areas of higher intensity. This result is similar to data reported for TASG by Shao *et al.* for live cell imaging of SGs, where findings indicated that the dye was mainly restricted to SG cores.^18^ In the present case as the peptide associates with rG4, it likely remains associated with these structures in the SG giving rise to the punctuated distribution around the SG.

### Immunostaining with stress granule markers

**Fig. 5:**
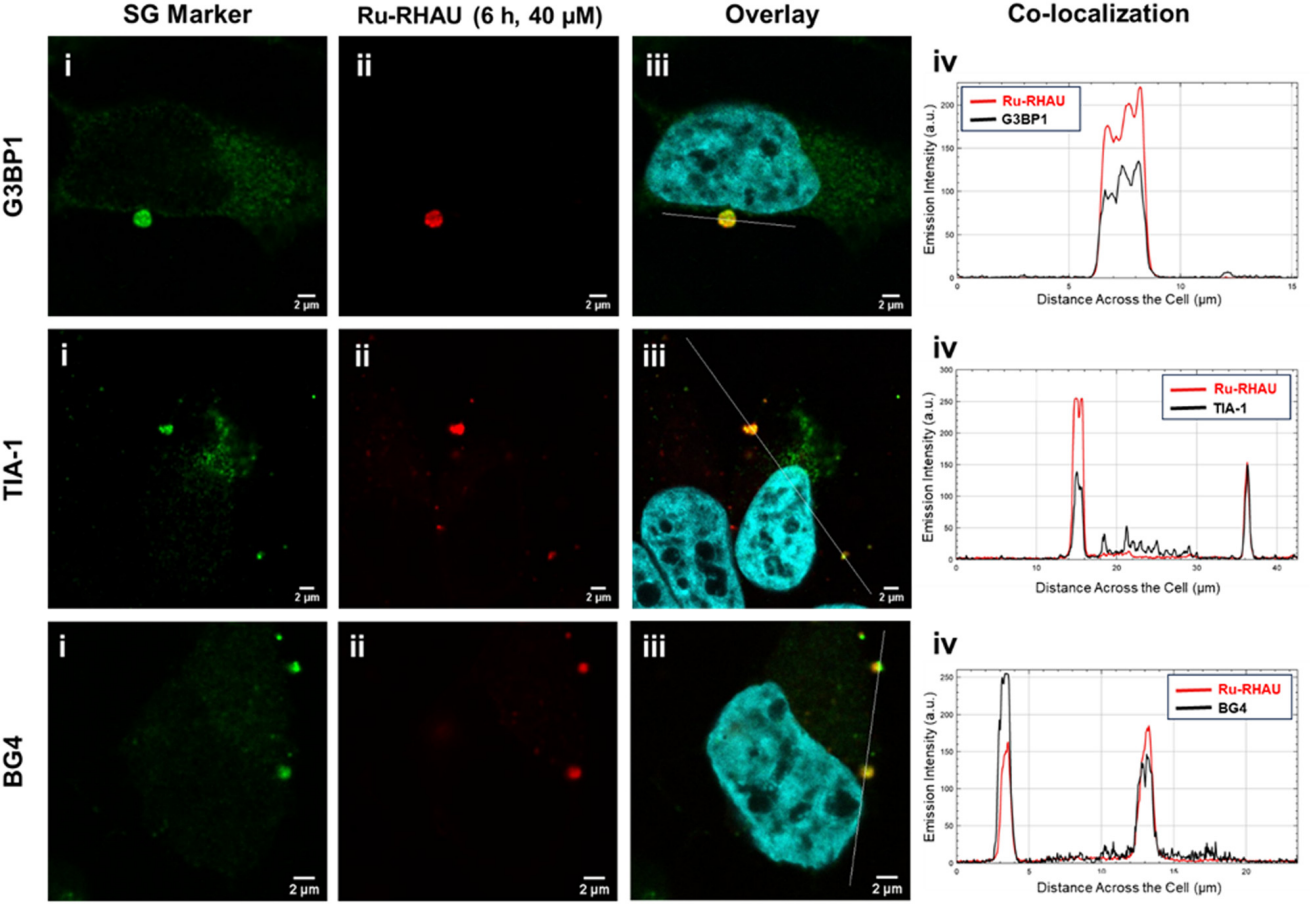
Confocal imaging of (i) G3BP1, TIA-1 or BG4 antibodies visualized using AlexaFluor 647 in fixed HeLa cells stained with (ii) Ru-RHAU at 40 μM for 6 h. The nucleus is indicated by Hoechst 33342 in (iii) the overlay images of (i) and (ii). The white lines indicate a selected region of interest to generate the corresponding co-localization graphs (iv). Co-localization imaging of Ru-RHAU at 25 μM, 24 h with G3BP1, TIA-1 or BG4 can be found in Fig. S21 (ESI†). Scale bars measure 2 μm.

To validate that Ru-RHAU is localized specifically to stress granules, immunostaining was carried out. There are many proteins found in SGs, however G3BP1 and TIA-1 have been the most widely studied.^51,52^ Reports suggest the interaction of G3BP1, an rG4-binding protein, and rG4s play a role in SG formation.^10^ Antibodies tagged with AlexaFluor-647 were employed to stain the SG localizing proteins, G3BP1 and TIA-1. We used the AlexFluor-647 tagged G3BP1 and TIA-1 to confirm the assembly of SGs in HeLa cells incubated with Ru-RHAU at both 6 h (40 μM) and 24 h (25 μM) incubation times. Strong co-localization of the larger foci of Ru-RHAU with G3BP1 and TIA-1 was observed (Fig. 5) supporting the idea that Ru-RHAU is capable of initiating SG assembly. Control studies were performed in HeLa cells using the SG markers under both normal conditions where there were no SGs in the cells and on heat shock induced SGs (42 °C, 1 h) to confirm the reliability of the G3BP1 and TIA-1 antibodies in detecting SGs under the imaging conditions used in this work. These controls validate the accuracy of SG detection observed in the presence of the dye (Fig. S21, ESI†).

### Ru-RHAU binds to rG4s in stress granules

As SG condensates contain rG4s bound by G3BP1 in response to cellular stress, and endogenous rG4s are enriched within SGs, we wanted to confirm that the Ru-RHAU SG labelling is associated with its binding to rG4s in SGs.^10^ To confirm this, co-localization of Ru-RHAU with immunofluorescence staining of BG4 was examined. BG4 is a single-chain variable fragment (scFv) antibody that has high affinity for both DNA and RNA G4s. It is not permeable and does not usually fold effectively in live mammalian cells, thus we carried out the study in fixed HeLa cells.^53^ HeLa cells treated with Ru-RHAU (40 μM, 6 h or 25 μM, 24 h) were fixed and then stained with anti-G4 antibody, BG4 (Merck). In the BG4 channel, staining can be seen throughout the entire cell with notable foci (approx. 2 μm) in the cytoplasm. The increased intensity of BG4 in comparison to control cells not treated with Ru-RHAU is striking, indicating Ru-RHAU role in SG generation.

Based on confocal imaging, strong co-localization is evident between BG4 and Ru-RHAU in the cytoplasm which is consistent with rG4 binding. This is further confirmed in the co-localization graph shown in Fig. 5. The co-localization with BG4 in combination with the stabilization of rG4 by Ru-RHAU and its evident ability to induce SGs, supports previously reported association between rG4s and SGs.^4^ It is important to note however, that while G3BP1 and TIA-1 are stress granules proteins, BG4, recognises G-quadruplex and so we cannot exclude the possibility that some of the structures co-labelled by Ru-RHAU and BG4 are p-bodies in addition to SGs, since both contain mRNA.

### Stress granule disassembly

Recovery of SGs following exposure to stressors such as sodium arsenite, hydrogen peroxide and heat shock have been reported to occur between 60 and 120 minutes after stressor removal. To test recovery of live HeLa cells post Ru-RHAU treatment, cells were treated with Ru-RHAU for 6 h (40 μM) or 24 h (25 μM) and imaged every 15 minutes for a total of 150 minutes (Fig. S29 and S30, ESI†) after the removal of Ru-RHAU. Imaging over time suggests the shorter incubation of 6 h enables the formation of early stage SGs, most of which were observed to disassemble over time following removal of Ru-RHAU. Longer, 24 h incubation with Ru-RHAU resulted in the formation of some large pathological, persistent SGs that were observed not to disassemble within a period of 150 minutes after the removal of the Ru(ii) complex. Pathological SGs have been implicated in the development of various cancers including breast and lung.^54^ The phase contrast images (Fig. S29 and S30, ESI†) show changes in cell morphology over time and indicate that, as the SGs disassemble, cell damage and ultimately cell death occurs. Cell death as a response to SG disassembly is thought to occur from the release of caspase proteins within the granules and initiation of apoptosis.^55^

### Toxicity of Ru-RHAU in HeLa cells

Identifying the factors that contribute to SG persistence may reveal potential therapeutic targets for diseases associated with persistent or pathological SGs. For example, inhibition of SG formation or promotion of SG disassembly may sensitize cancer cells to chemotherapy and improve treatment outcomes. The prospect of developing a SG targeting therapeutic is thus appealing, particularly given their prevalence in chemotherapeutic resistance.^56,57^ To this end, MTT toxicity assays were performed in HeLa cells following the removal of Ru-RHAU after 6 h or 24 h incubations in the dark at 37 °C. The complex was not very toxic with an IC_50_ > 100 μM (6 h) and 59 ± 0.98 μM (24 h) which is significantly higher than the concentrations selected for imaging. Although some large SGs assemble after 6 h incubation, the shorter incubation period may support a reduced level of cellular stress during the MTT assay incubation following the removal of Ru-RHAU, resulting in the higher dark IC_50_ of >100 μM. In contrast, a 24-hour incubation period likely exacerbates stress responses, leading to a lower IC_50_ of 59 ± 0.98 μM. To determine the mechanism of cell death, autophagy and apoptosis assays were completed with cells treated with 50 and 60 μM Ru-RHAU. The assay results indicates that a 24 h incubation with Ru-RHAU induces cell death through apoptosis, confirmed by the FLICA polycaspase assay where 60–62% of cells were apoptotic relative to control cells treated with Staurosporine. Furthermore, it was confirmed that the cells are not undergoing autophagy, with <3% increase in autophagic vacuoles relative to untreated control cells, in comparison to an 81% increase in autophagy induced control cells treated with Rapamycin and Chloroquine. In contrast, after a 6 h incubation some cells exhibited signs of autophagic cell death, with approximately 20% of cells displaying markers of autophagy. Interestingly, comparable levels of apoptosis were observed both after 6 h and 24 h incubation periods where approximately 57–58% of cells treated with Ru-RHAU showed apoptotic features after a 6 h incubation. The FLICA polycaspase assay detects multiple activated caspases, including caspase-3 and -7 which have been linked to apoptosis.^58^

As changes to cell morphology indicate decreased cell health during SG disassembly, we were interested to study the dark toxicity after 2 h removal of Ru-RHAU in cells that had been treated for 24 h. Allowing SG disassembly to begin (2 h) prior to the MTT cell viability assay resulted in a significant change in the dark IC_50_ of the cells after a 24 h incubation from 59 ± 0.98 μM to 28 ± 4.33 μM. This observation indicates that the SGs may provide protection to the cells while Ru-RHAU is present, however once the complex is removed, the SGs begin to disassemble and cell viability decreases. It is important to note however, that a 24 h incubation with Ru-RHAU is an exceptionally long time for the cells to be under stress, therefore it is not surprising that we see dark toxicity over these time windows.

Ruthenium complexes have been extensively investigated as potential phototherapeutics.^59–64^ To explore the phototoxicity of Ru-RHAU, the impact of photoirradiation on cell health after Ru-RHAU induced SG formation was evaluated. Cells were irradiated at a total dose of 5 ± 0.29 J cm^−2^ (0.5 h at 2.63 ± 0.16 mW cm^−2^) using a 470 nm LED under normoxic conditions after the removal of Ru-RHAU. Light IC_50_ values of 14 ± 0.19 μM and 15 ± 0.16 μM were recorded after an incubation period of 6 h and 24 h respectively. The similarity in light IC_50_ values indicate that prolonged incubation does not impact the phototoxic effect of Ru-RHAU upon irradiation, this is not surprising given the rapid uptake of the conjugate which is complete within 6 hours. The results suggest the potential of SGs as a target for phototherapy. It is important to note that imaging was performed under significantly lower irradiation power density than phototoxicity studies and the absence of DRAQ7 uptake (3 μM) under imaging conditions confirmed cells remain healthy.

### Ru-RHAU as a probe for stress granules induced by external stress stimuli in live cells

To consider Ru-RHAU as a probe for SG formation under application of external stressors, we examined it under stressor conditions at lower concentration and shorter incubation times than reported *vide supra*.

HeLa cells were incubated with Ru-RHAU at 10 μM for 3 or 6 h. A 10 μM concentration was selected as it was well below the concentration at which the Ru-RHAU complex induces SGs. After the incubation with Ru-RHAU, the contact solution (Ru in media) was removed from the cells and they were treated with heat shock (42 °C, 1 h) in phenol red free media. Images were acquired immediately and localization in SGs was observed.

**Fig. 6:**
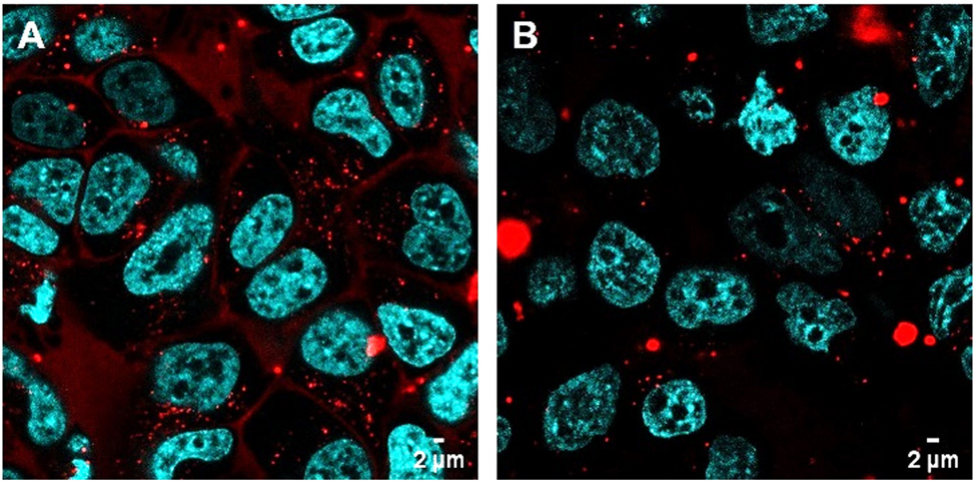
HeLa cells treated with Ru-RHAU (10 μM) (A) at *t* = 0 and (B) *t* = 30 min after the addition of Ru-RHAU. SGs were induced by heat shock (42 °C, 1 h) prior to the addition of Ru-RHAU. Scale bars measure 2 μm.

To determine the rate of uptake of Ru-RHAU (10 μM) into the SGs, untreated HeLa cells were subjected to heat shock and the uptake of Ru-RHAU was imaged every 15 minutes from *t* = 0 *i.e.* from the addition of Ru-RHAU. Confocal imaging was completed using a heated stage to ensure no SG disassembly and rapid accumulation of the dye into SGs was observed, as shown in Fig. 6. The concentration and time dependence of Ru-RHAU induced SG assembly means the dye can be used for both live cell imaging of SGs induced using the Ru-RHAU complex itself, or alternatively at lower concentrations and shorter incubation times, it can be used as a dye for live cell imaging of SGs induced through external stimuli.

## Conclusions

We report on the first rG4 targeted Ru(ii) complex as a powerful probe to enable stimulation and visualization of SGs in live cells. Ru-RHAU, comprises of a RHAU helicase derived peptide conjugated to [Ru(bpy)_2_(pic)]^2+^ where is pic is 2-(4-carboxyphenyl)imidazo[4,5-*f*][1,10]phenanthroline.

It is water soluble and permeable to live cells through a passive uptake mechanism. Ru-RHAU was used to successfully visualize SGs in live cells, inducing them in a concentration and time dependent manner and is to the best of our knowledge, the first reported dye to do this. Additionally, at lower concentrations and shorter incubation times, this dye can be used to visualize the dynamics of SGs induced using external stress stimuli. G4 binding was established in solution with several G4 sequences, where the highest binding affinity was shown for CMYC and significant stabilization was observed for NRAS, an rG4 sequence (*T*_m_ increased by 26 °C). G4 binding was confirmed in fixed HeLa and MCF-7 cells by immunostaining studies with BG4. SG assembly was confirmed by fixed cell imaging of Ru-RHAU treated cells with SG markers G3BP1 and TIA-1 where exceptional overlap was displayed in the SGs. As Ru-RHAU can be applied for imaging SGs in both live and fixed cells, it offers an opportunity to overcome the current limitations of commercially available dyes, which are restricted to visualizing SGs in fixed cells only. Furthermore, Ru-RHAU displayed mild cytotoxicity after a 24 h incubation, however with inclusion of a 2 h window to allow for SG disassembly, there is a significant increase in cytotoxicity indicating the presence and disassembly of SGs could play a critical role in cytotoxicity. Additionally, Ru-RHAU exhibited high phototoxicity after both 6 h or 24 h incubation, suggesting SGs are prospective phototherapeutic targets with potential for overcoming therapy resistant cancer. Overall, these results indicate Ru-RHAU is a promising probe for visualizing SG dynamics in a live cell environment. Targeting SGs or the pathways involved in their formation and persistence is emerging as a potential strategy to overcome cancer therapy resistance that such photoactive complexes may be applied to.

Future work will focus on studies of this probe in additional cell lines, such as neuronal cells and could enhance the understanding of the role of SGs in disease pathology.

## Author contributions

R. C. C. performed all cellular work and co-wrote the manuscript. L. H. prepared and characterized Ru-RHAU, performed all DNA and RNA studies and co-wrote the manuscript. T. E. K. conceptualized the project and secured funding for this research, supervised the project and edited the manuscript.

## Conflicts of interest

There are no conflicts to declare.

## Supplementary Material

CB-OLF-D5CB00008D-s001

## Data Availability

The data supporting this article have been included as part of the ESI.†
