# Supplementary material for: Visualizing stress granule dynamics with an RNA guanine quadruplex targeted ruthenium(ii) peptide conjugate
Source: RSC Chem Biol. 2025 Jun 19;6(9):1403–13. doi: 10.1039/d5cb00008d (PMC12188320; doi:10.1039/d5cb00008d)
Supplement: CB-006-D5CB00008D-s001 [file CB-006-D5CB00008D-s001.pdf]

Supplementary Information (SI) for RSC Chemical Biology.
This journal is © The Royal Society of Chemistry 2025

Electronic Supporting Information

Visualizing Stress Granule Dynamics with an RNA Guanine Quadruplex Targeted Ruthenium(II) Peptide Conjugate

Rhianne C. Curley‡, Lorcan Holden‡, and Tia E. Keyes*

School of Chemical Sciences, Life Sciences Institute, Dublin City University, Dublin 9, Co. Dublin, Ireland

* *Corresponding author*: *tia.keyes@dcu.ie*

Synthesis and Characterization

Reagents

Chemicals were purchased either from Sigma-Aldrich (Merck) or Fluorochem and used without further purification. Oligonucleotides were purchased from Eurofins Genomics and used without further purification. Peptides were purchased from Celtek Peptides and provided at >95% purity as determined by HPLC.

[Ru(bpy)$_2$Cl$_2$]

[Ru(bpy)$_2$Cl$_2$] was synthesised as previously reported by Sullivan *et al*. where $RuCl_3$ was dissolved in 10 mL of anhydrous DMF with 2,2-bipyridine and lithium chloride.[1] The solution was refluxed for 8 h under N_2, followed by precipitation in acetone, being filtered and washed. Purification by silica chromatography (MeOH/DCM) yielded fractions that were evaporated under pressure to yield pure [Ru(bpy)$_2$Cl$_2$] as a dark purple solid. ^{1}H NMR (600 MHz, DMSO-d$_6$): 9.20 (d, 2 H), 8.64 (d, 2 H), 8.48 (d, 2 H), 8.11 (t, 2 H), 7.46 (2H) 7.35 (d, 2 H), 7.15 (d, 2 H), 7.05 (2 H).

Pic-COOH

To synthesize Pic-COOH the method developed previously by the Keyes group was used.[2] Phendione, carboxybenzaldehyde and ammonium acetate were added to a RBF in acetic acid and refluxed for 6 h. The solution was cooled, and water was added to precipitate the product before filtering and washing with water, methanol and diethyl ether. The ligand was used without further purification. ^{1}H NMR (600 MHz, DMSO- d$_6$): 13.91 (s, 1 H), 12.70 (s, 1 H), 9.03 (dd, 2 H), 8.90 (dd, 2 H), 8.38 (d, 2 H), 8.17 (d, 2 H), 7.82 (q, 2 H).

[Ru(bpy)$_2$(Pic-COOH)]$^{2+}$

Pic-COOH was easily complexed to [Ru(bpy)$_2$Cl$_2$] by refluxing in MeOH before purification on silica column with an 80/20/1 (MeCN/ H_2O/ 20% w/v KNO_2) mobile phase. Fractions of the purified product were collected and combined before evaporation under vacuum. [Ru(bpy)$_2$(Pic-COOH)]$^{2+}$ was dissolved in minimal acetone before precipitating the complex as a PF_6 salt by stirring in an aqueous ammonium hexafluorophosphate solution. A portion of [Ru(bpy)$_2$(Pic-COOH)]$^{2-}$ was converted to a chloride salt for use as a parent complex in photophysical and biophysical studies. Conversion to a chloride salt was achieved by dropwise addition of the complex dissolved in a minimal quantity of acetone to a stirring solution of TBAC in acetone. The chloride precipitate was filtered and washed with copious quantities of acetone. ^{1}H NMR (600 MHz, DMSO- d$_6$) 9.05 (s, 2 H), 8.54 (d 2 H) 8.50 (d, 2 H), 8.38 (d, 2 H), 8.21 (d, 2 H) 8.10 (t, 2 H), 8.00 (m, 4 H), 7.85 (d, 2 H), 7.74 (q, 4 H), 7.61 (d, 2 H), 7.45 (t, 2 H), 7.22 (t, 2 H).

Ru-RHAU

Ru-RHAU was synthesized using conjugation methods previously reported in our group.[3] $[Ru(bpy)_2(Pic\text{-}COOH)]^{2+}$ (5 mg), NHS (5eq) and DCC (5eq) were added to a RBF with 10 mL of anhydrous MeCN and stirred. Formation of the NHS intermediate was monitored by TLC, when complete the solution was evaporated and redissolved in 1 mL DMF. The RHAU peptide sequence (1.1eq) was dissolved in 2 mL of PBS and added to the RBF. The solution was stirred overnight. After 18 h the solution was purified using a C18 silica prep plate. The band identified as the conjugated product, Ru-RHAU, was isolated. Ru-RHAU dissolved by MeCN and filtered through a bed of celite before evaporation under pressure until approximately 1 mL of solvent remained. Ru-RHAU was then yielded as PF_6 salt by slow addition to stirring water saturated with ammonium hexafluorophosphate. The dark red ppt. was filtered and washed with water followed by diethyl ether. Conversion to a chloride salt was completed by dissolving the complex in minimal MeCN before stirring overnight on amberlite chloride exchange resin. The complex was evaporated to dryness, dissolved in MeOH and filtered in order to remove resin. After filtration, the Ru-RHAU solution was evaporated to dryness yielding the final product (2.4 mg, 13%). ^1H NMR (600 MHz, MeOD/D_2O) 8.78 (6H), 8.22 (t, 2H), 8.16 (t, 2H), 8.11 (d, 1H), 8.08 (3H), 8.01 (dd, 2H), 7.98-7.94 (5H), 7.7 (d, 1H), 7,64 (m, 7H), 7.58 (4H) 7.44 (2H) 7.35 (1H), 5.45 (2H), 4.62 (4H), 3.68-4.30 (16H), 3.58 (7H), 3.44 (4H), 3.32 (3H), 3.11 (7H), 3.05 (1H), 2.95 (5H), 2.51 (2H), 2.3 (2H), 2.0 (3H), 1.86 (7H), 1.77 (2H), 1.7 (6H), 1.55 (9H), 1.35 (29 H), 1.14 (15H), 0.88 (19H). m/z calculated = 3382.6494, found = 3382.5083.

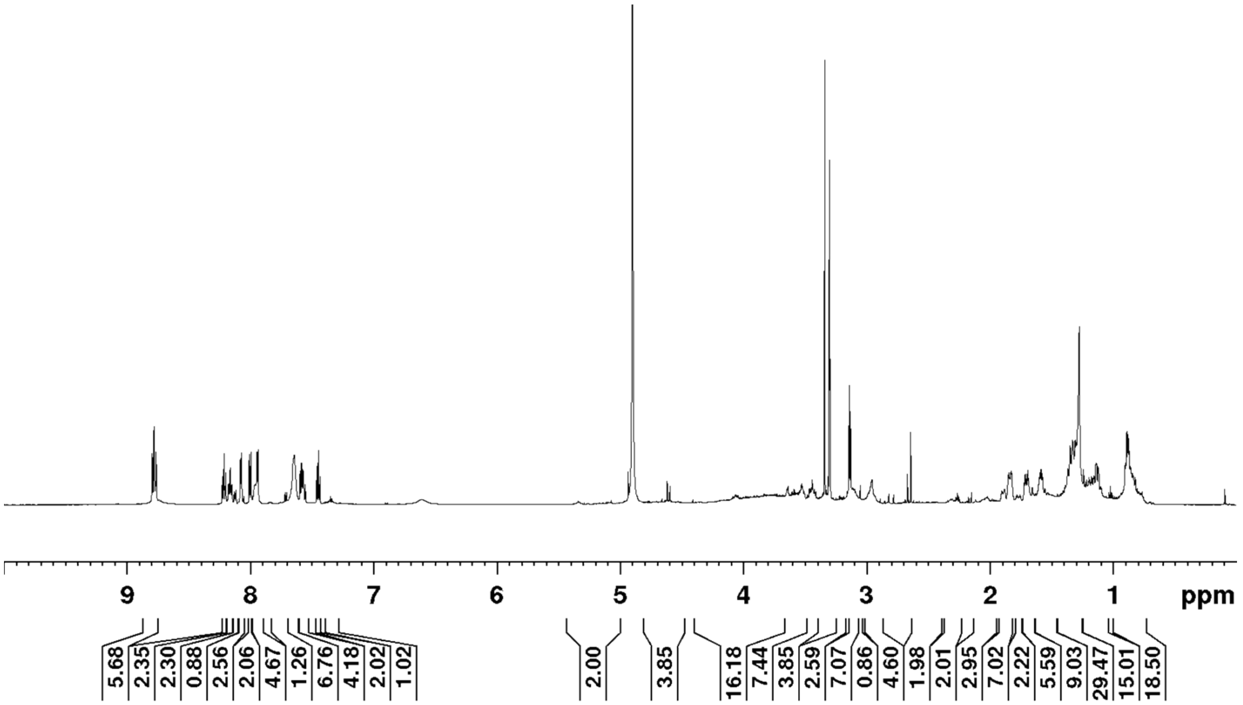

Figure S1. ^1H NMR of Ru-RHAU (CD_3OD, D_2O).

HPLC

Chromatograms of the three compounds were obtained using the same method reverse phase method, applying a gradient system H_2O/MeCN with 0.1% TFA. Figure S2 A shows representative chromatograms for top, NH_2HexRHAU peptide precursor, middle, $[Ru(bpy)_2(PIC\text{-}COOH)]^{2+}$ and bottom, the peptide conjugate monitoring at 280 nm. Peak detection was recorded at 280 nm and 450 nm using a photodiode array detector. Figure S2 B shows the UV-Vis spectra of each peak recorded on diode array.

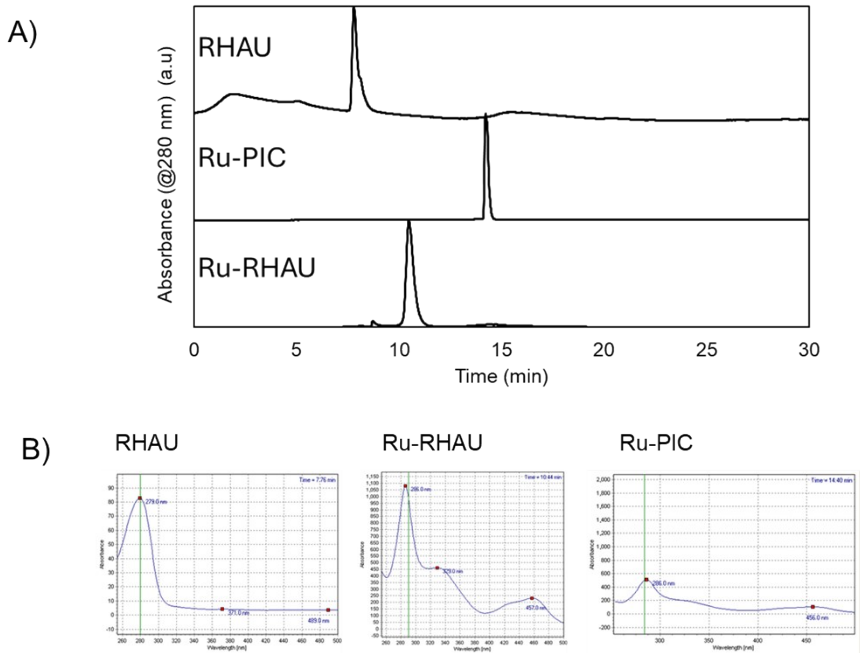

Figure S2. **(A)** HPLC chromatograms of RHAU peptide, Ru-PIC and Ru-RHAU. **(B)** Absorbance spectra gathered from the PDAD from 280 to 500 nm.

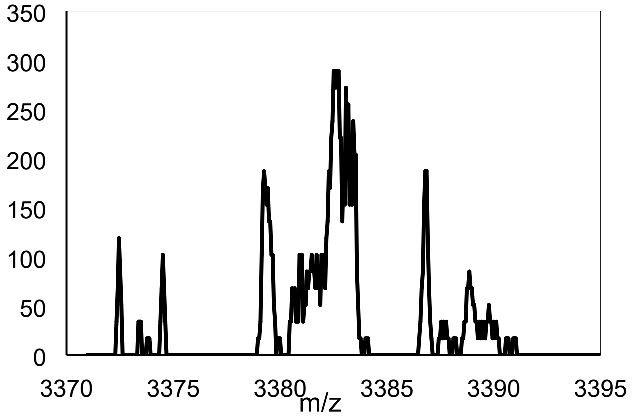

Figure S3. Mass Spec of Ru-RHAU expansion of the 1+ region.

Spectroscopic Studies

Absorbance measurements were made using a Jasco V670 spectrophotometer, data was manipulated using Jasco Spectra Manager software and MS Excel. Luminescence studies were performed on a Varian Cary Eclipse Fluorimeter. TCSPC measurements were made using a PicoQuant FluoTime 100 Compact TCSPC system with a 450 nm laser from a PicoQuant PDL800-B Source and an external Thurlby Thander TGP110 10 MHz pulse generator. TCSPC spectra was collected up to 10000 counts, and decays were analysed and fit with PicoQuant Fluofit software

Stock concentrations of Ru-RHAU and Ru-PIC were stored at -20 °C as Cl^- salts in deionized water at a concentration of 1 mM and 4 mM respectively.

Oligonucleotides were purchased from Eurofins Genomics and stored at -20 ^oC. Preparation of G4s were performed similarly to previous reports. A KPi buffer (10 mM potassium phosphate, 100 mM KCl) was used to anneal the quadruplex for all absorption, fluorescence and TCSPC measurements. Quadruplexes were annealed at 95 ^oC for 5 mins before cooling slowly to room temperature. DNA fluorescence and absorbance titration experiments were performed in tandem using a stock concentration of 1mM quadruplex strand concentration. RNA fluorescent and absorbance titration experiments were performed in tandem using a stock concentration of 100 µM quadruplex strand concentration.

CD melting studies were performed at an oligonucleotide concentration of 5 µM and measured over a window of 220 – 290 nm from 20 to 94 °C. The oligos KRAS, 22AG and Pu24T were annealed in KPi buffer. Due to the increased stability of CMYC, a lithium cacadolyte buffer was used (10 mM) with additional LiCl (90 mM). Circular dichromism measurements were performed on a Chirascan Series and data manipulation was performed using the Global 3 Analysis Software and MS Excel.

Table S1. Panel of oligonucleotides selected for testing.

Name	Secondary structure	Sequence 5′→3′	Ref.
22AG	Mixed hybrid	AGGGTTAGGGTTAGGGTTAGGG	[4]
KRAS	Parallel	AGGGCGGTGTGGGATAGGGAA	[5]
CMYC	Parallel	TGGGGAGGGTGGGGAGGGTGGGGAAGG	[6]
Pu24T	Parallel	TGAGGGTGGTGAGGGTGGGGAAGG	[4]
NRAS	Parallel	UGUGGGAGGGGCGGGUCUGGGUGC	[7]
ds26	Double Stranded	CAATCGGATCGAATTCGATCCGATTG	[8]
ctDNA	Double stranded		

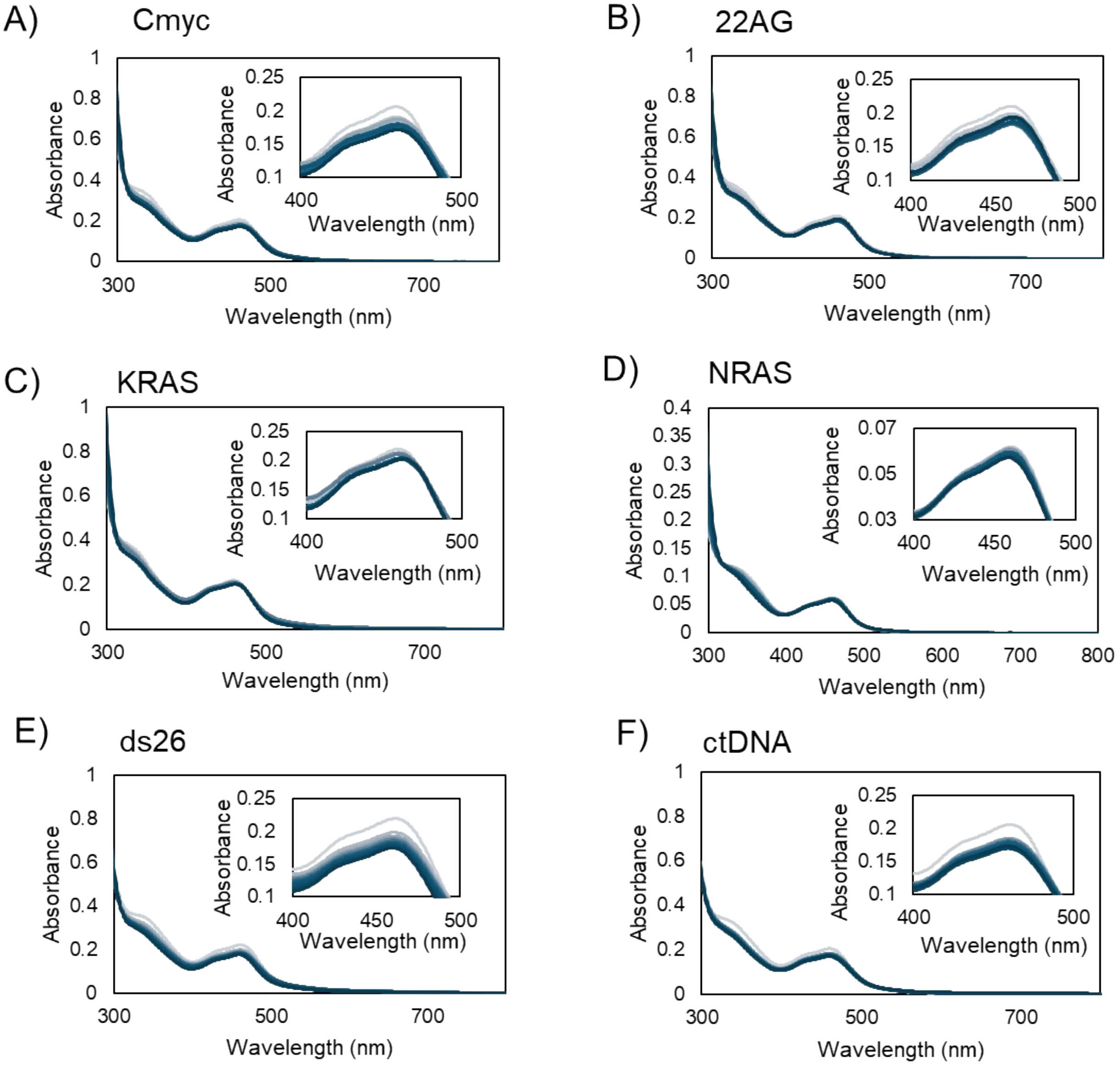

Figure S4. Representative absorption spectra obtained from a series of DNA titrations. Ru-RHAU (10 µM) was titrated with the DNA G4s CMYC, KRAS, 22AG (0- 30 µM) and the duplex DNA ds26 and ctDNA (0-50 µM base pairs). Ru-RHAU (2 µM) titration with the rG4 NRAS (0-6 µM) in RNase free buffer. Insets of all graphs show the expanded MLCT region from 400 to 500 nm.

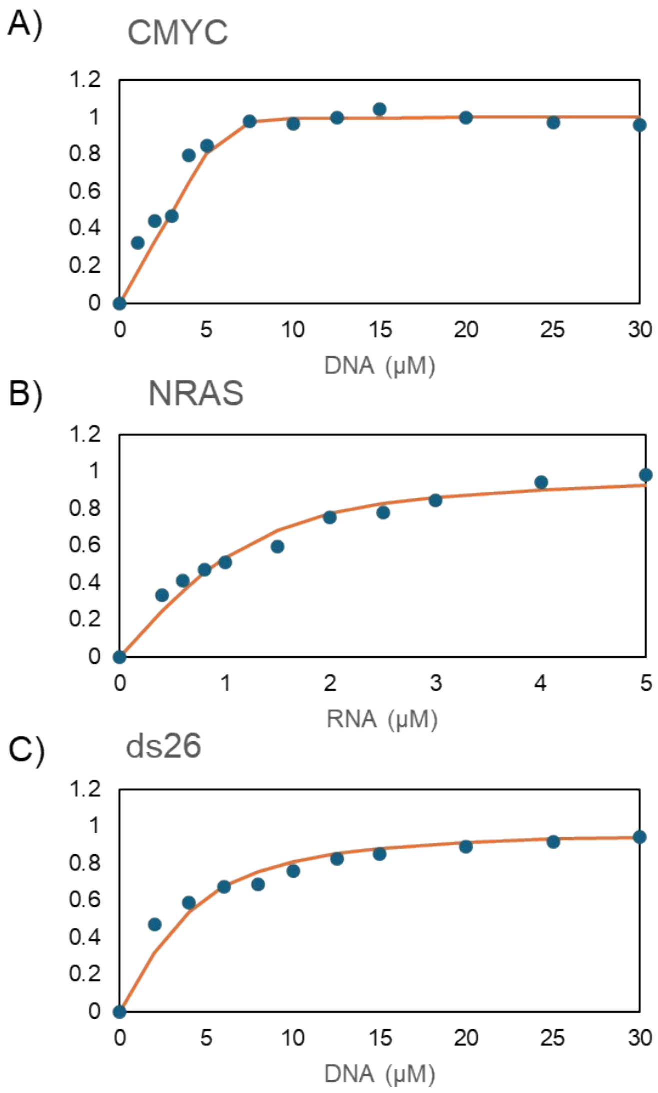

Figure S5. Representative absorption titrations of Ru-RHAU with CMYC, NRAS and ds26 and their respective binding curves.

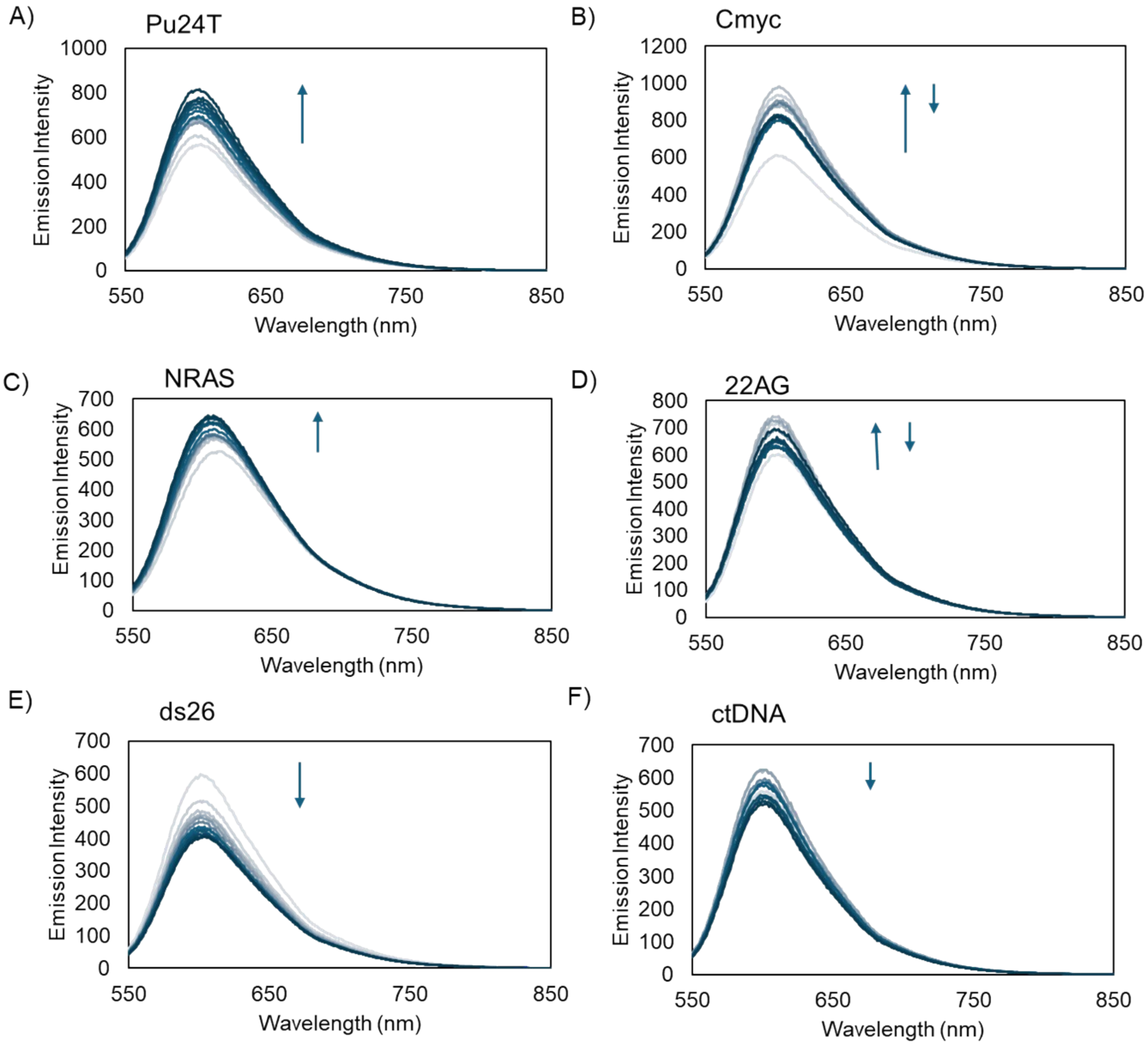

Figure S6. Representative emission spectra obtained from a series of DNA titrations. Ru-RHAU (10 µM) was titrated with the DNA G4s CMYC, KRAS, 22AG (0- 30 µM) and the duplex DNA ds26 and ctDNA (0-50 µM base pairs). Ru-RHAU (2 µM) titration with the rG4 NRAS (0-6 µM) in RNase free buffer. Ru-RHAU was excited at 490 nm. Arrows indicate the increase or decrease in emission intensity upon addition of oligonucleotides. A second arrow indicates a small decrease in intensity that occurs after reaching an emission maximum with the further addition of oligonucleotides.

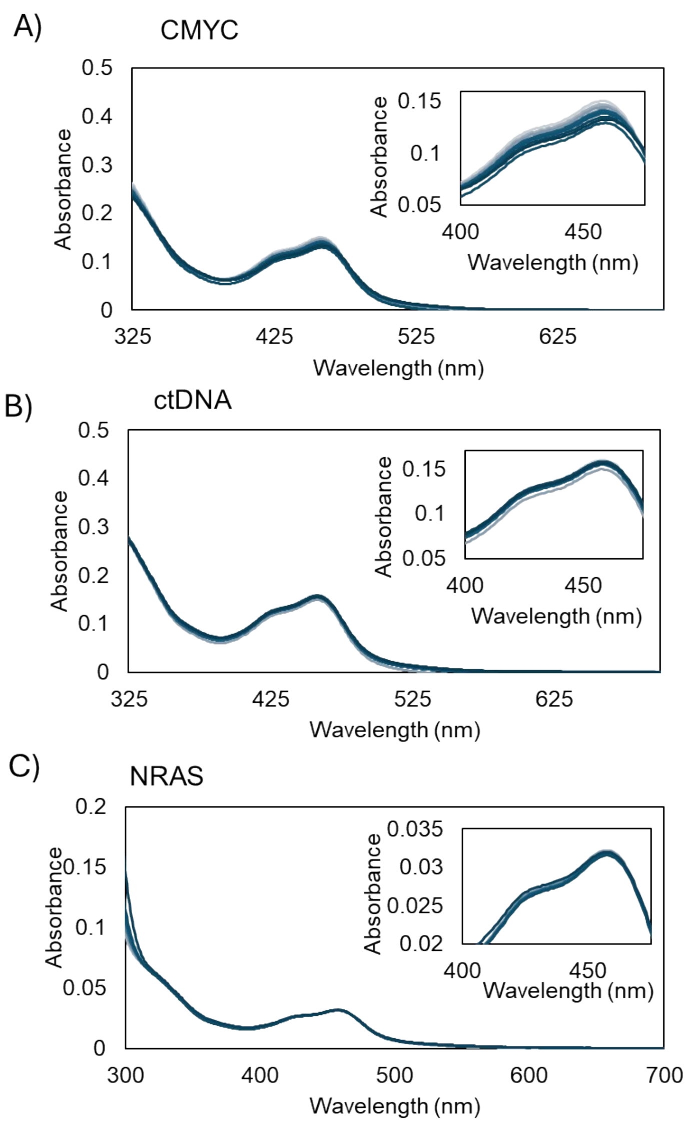

Figure S7. Representative absorption spectra of Ru-PIC (10 μM) when titrated with **(A)** CMYC (0-30 μM) and **(B)** ctDNA (0-50 μM). **(C)** Representative luminescence spectra of Ru-PIC (2 μM) when titrated with NRAS (0-6 μM). The inset figures show expanded MLCT regions from 400 to 500 nm.

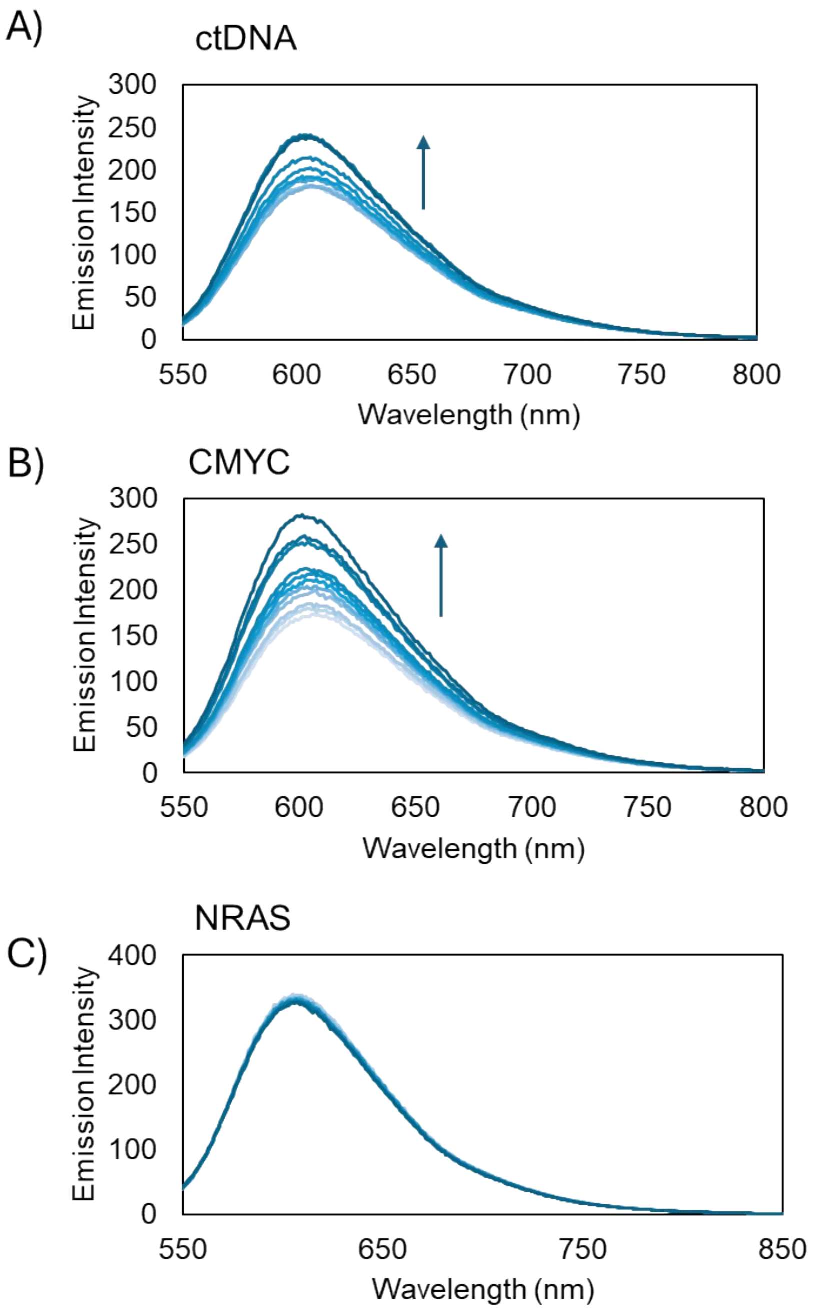

Figure S8. Representative luminescence spectra of Ru-PIC (10 µM) when titrated with **(A)** ctDNA (0-50 µM) and **(B)** CMYC (0-30 µM). Excitation at 490 nm (Slit widths 5 nm). **(C)** Representative luminescence spectra of Ru-PIC (2 µM) when titrated with NRAS (0-6 µM) (Slit widths 10 nm).

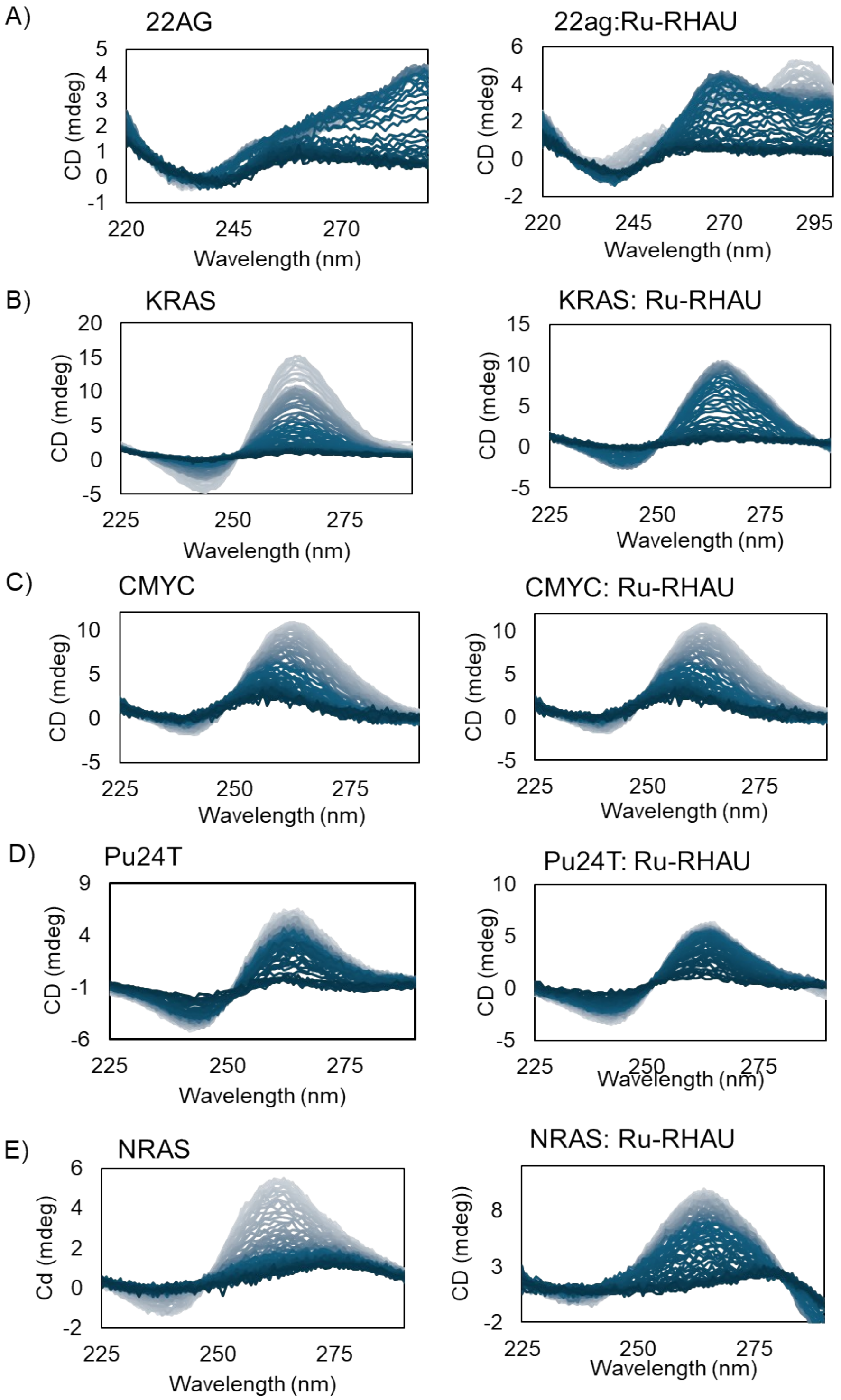

Figure S9. Melting curves of G4s at (5 μM) and when bound to Ru-RHAU (10 μM), **(A)** 22AG, **(B)** KRAS, **(C)** CMYC, **(D)** PU24T and **(E)** NRAS. Spectra are measured for every increase in 1 °C from 20 °C to 94 °C across a window of 225 nm to 290 nm.

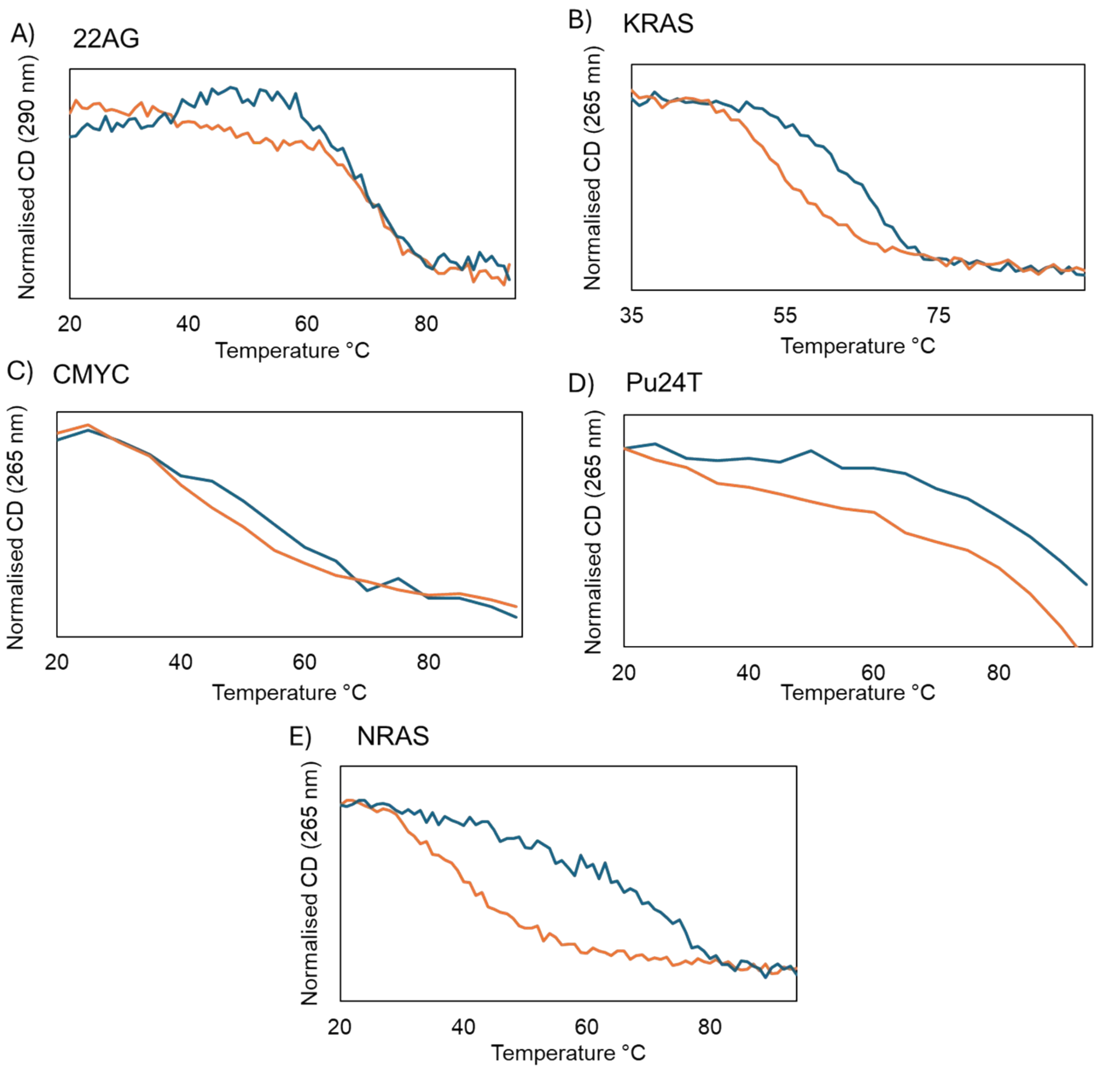

Figure S10. Comparison of thermal stability of G4s (5 μM) when unbound (orange) and bound (blue) to Ru-RHAU (10 μM).

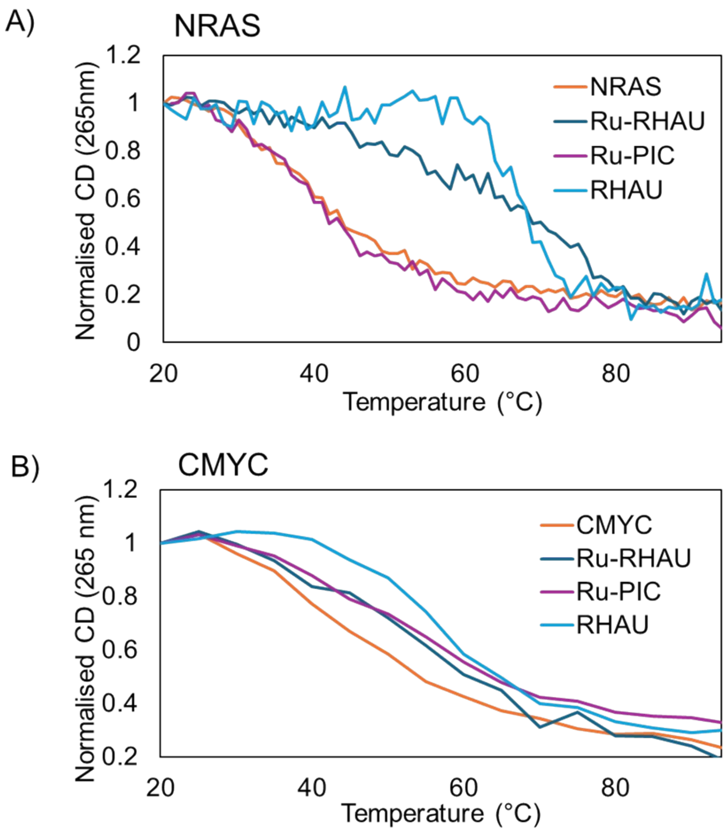

Figure S11. **(A)** Normalized changes in the CD signal of NRAS (μM) at 265 nm when bound to 2 equivalents of RHAU, Ru-PIC or Ru-RHAU (10 μM). **(B)** Normalized changes in the CD signal of CMYC (μM) at 265 nm when bound to 2 equivalents of RHAU, Ru-PIC or Ru-RHAU (10 μM). Increasing temperature from 20 °C to 94 °C at a rate of 1 °C/min.

Cellular Uptake and SG Assembly

HeLa cells were seeded at 1 × 10^5 cells/mL in 8 chamber slides (Ibidi, Germany) and allowed to grow for 24 h at 37 °C with 5% CO_2. Cells were treated with Ru-RHAU, by diluting a 1 mM stock solution of the complex in cell culture media. After the desired incubation time e.g. 24 h, the Ru-media solution was removed, and the cells washed twice with 1X PBS (Gibco). The cells were imaged in phenol red free DMEM (Gibco) supplemented with L-glutamine and 10% FBS. Cells were imaged using a Leica TCS SP8 DMi8 confocal microscope (63× or 100x oil immersion objective lens) with a heated stage at 37 °C. Ru-RHAU was excited at 488 nm and the emission range set to between 550 and 800 nm. Uptake was assessed after treating the cells with Ru-RHAU under the following conditions: 10, 15, 25, 30, 35, 40, 50 and 100 µM at 6 h incubation, 25, 30, 35, 40 µM for 8 h incubation and 10, 15, 25, 30, 40, 50 µM for 24 h. DRAQ7 (3 µM) was added each well prior to imaging to determine if the cells were damaged under the imaging conditions being tested. MCF-7 cells were seeded at 1 × 10^5 cells/mL in 8 chamber slides and allowed to grow for 48 h at 37 °C with 5% CO_2 and treated with Ru-RHAU at 40 µM (6 h) or 25 µM (24 h).

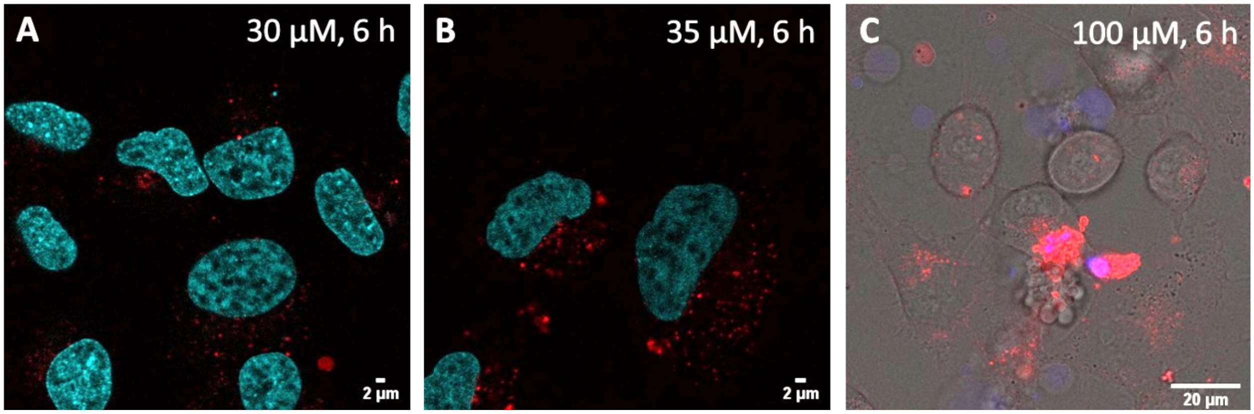

Figure S12. Confocal images of **HeLa** cells after a 6 h incubation with Ru-RHAU at **(A)** 30 µM, **(B)** 35 µM and **(C)** 100 µM.

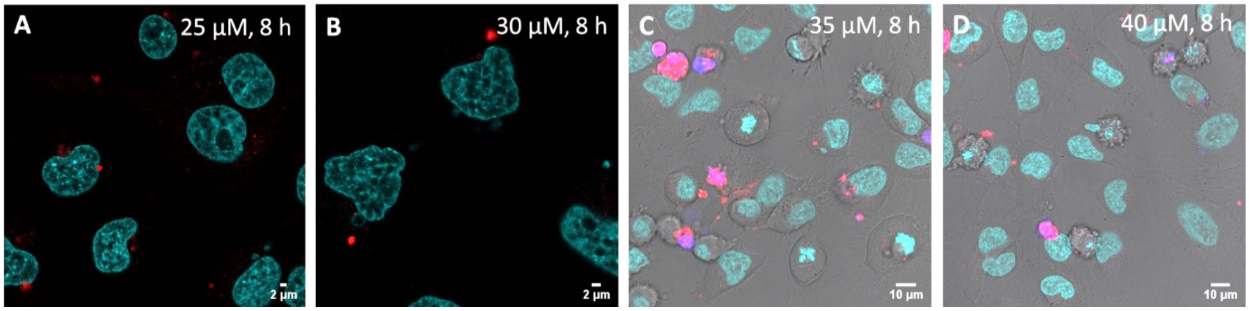

Figure S13. Confocal images of **HeLa** cells after an 8 h incubation with Ru-RHAU at **(A)** 25 µM, **(B)** 30 µM and **(C)** 35 µM and **(D)** 40 µM. 63x oil lens.

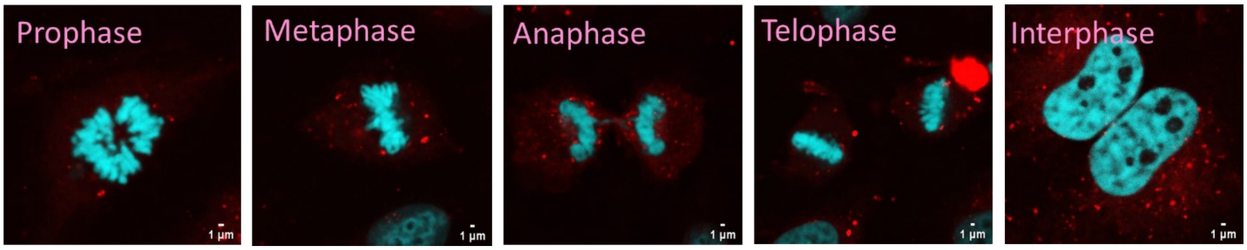

Figure S14. Confocal images of fixed HeLa cells with Ru-RHAU (40 µM, 6 h) at various cell cycle phases. Hoechst 33342 stains the nucleus**.**

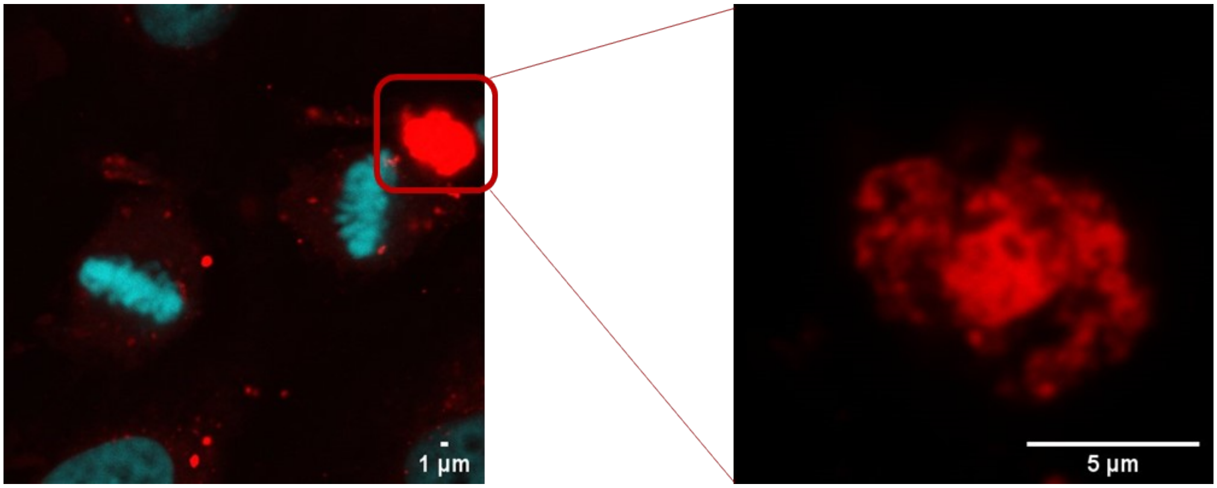

Figure S15. Confocal image of **HeLa** cells in telophase with corresponding zoomed image of large SG imaged at lower intensity to allow for better visualization of the SG contents.

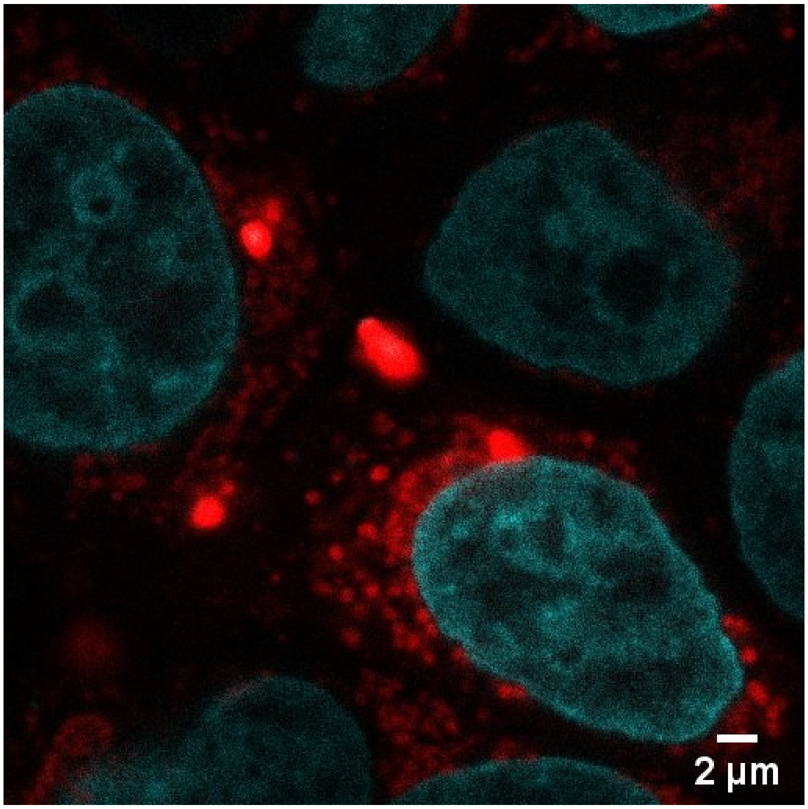

Figure S16. Confocal image of Ru-RHAU (40 µM, 6 h) in live **MCF-7** cells. Hoechst 33342 (cyan) indicates the nucleus. 100x oil.

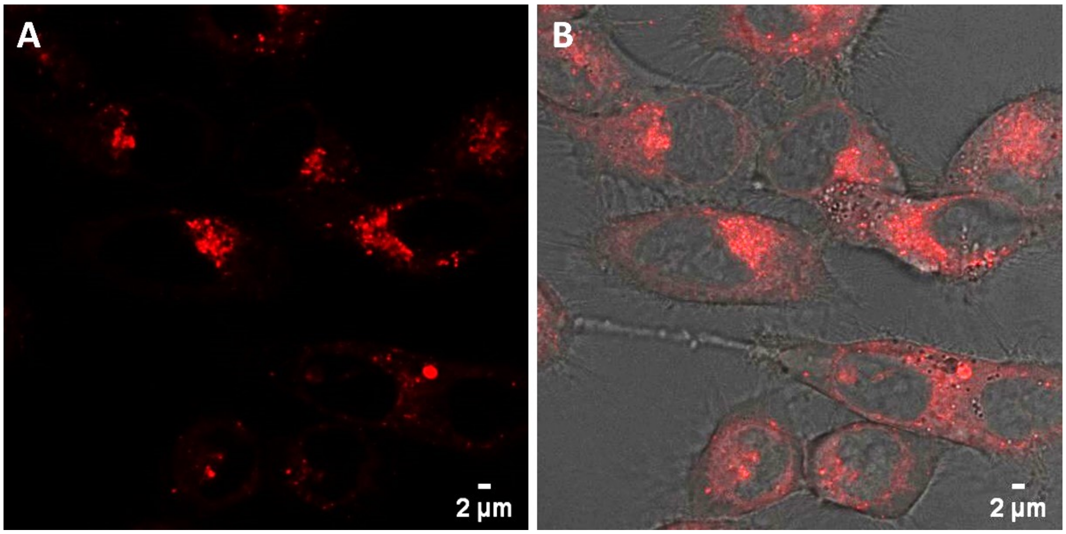

Figure S17. **(A)** Live **HeLa** cells pre-treated with Oligomycin and 2DG prior to Ru-RHAU (25 uM, 24 h) and **(B)** the overlay with phase contrast channel.

Localization in Live HeLa Cells

Co-localization studies were performed in HeLa cells with LysoTracker Deep Red lysosomal dye (100 nM, 1 h) with a WLL at 647 nm and emission collected between 665-675 nm.

HeLa cells were seeded in 8 chamber slides (ibidi) at 1 x 10^5 cells/mL and allowed to grow and attach overnight before being treated with Ru-RHAU at 40 µM for 6 h or 25 µM for 24 h. After the incubation period the cells were washed twice with 1X PBS and imaged in phenol red free media using a 100x oil immersion lens unless stated otherwise. Ru-RHAU was excited at 488 nm and emission collected between 580-645 nm. Co-localization analysis was performed using ImageJ software.

Concentration of Ru-RHAU (µM)	Incubation time (h)	Commercial Dye	Organelle	Pearson's Coefficient r =
25	24	LysoTracker Deep Red	Lysosomes	0.52 ± 0.08
40	6			0.47 ± 0.06

Table S2. Co-localization results of Ru-RHAU with LysoTracker Deep Red in live HeLa cells.

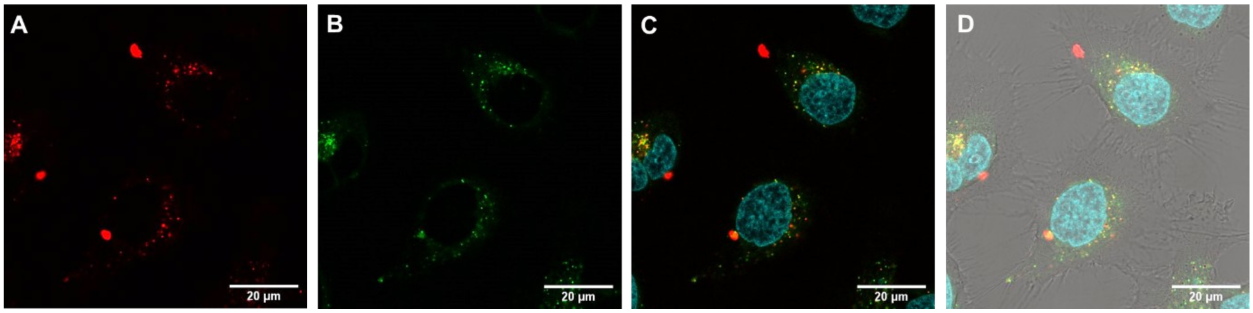

Figure S18. Confocal imaging of **(A)** Ru-RHAU (25 µM, 24 h), **(B)** LysoTracker Deep Red (100 nM, 45 min) and **(C)** overlay of A and B with Hoechst 33342 nuclear stain (1 µg/mL, 15 min) and **(D)** phase contrast in live **HeLa** cells. 100x oil.

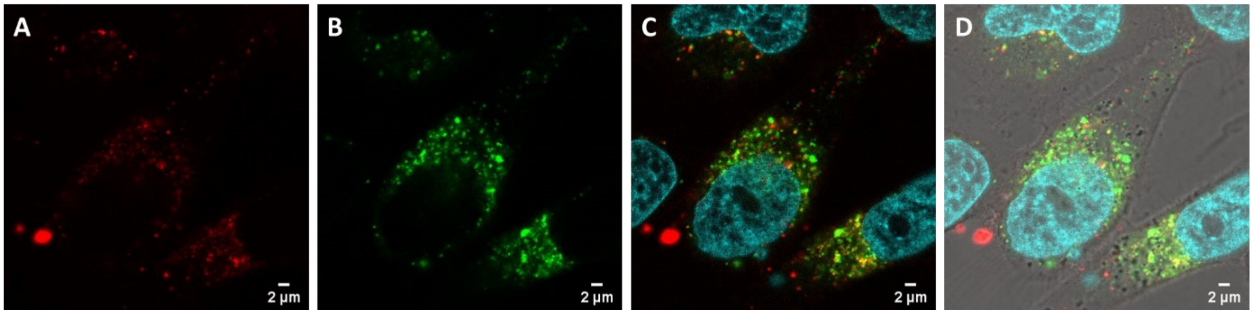

Figure S19. Confocal imaging of **(A)** Ru-RHAU (40 µM, 6 h), **(B)** LysoTracker Deep Red (100 nM, 45 min) and **(C)** overlay of A and B with Hoechst 33342 nuclear stain (1 µg/mL, 15 min) and **(D)** phase contrast in live **HeLa** cells. 100x oil.

Super-Resolution Imaging

Live and fixed HeLa cells were prepared as per routine cell imaging studies for super-resolution imaging using either the Zeiss LSM 980, Airyscan 2 (Figure 4) and for routine confocal imaging using the Leica DMi8 confocal microscope to depict Ru-RHAU intensity in SGs as a comparison to the Airyscan 2 imaging.

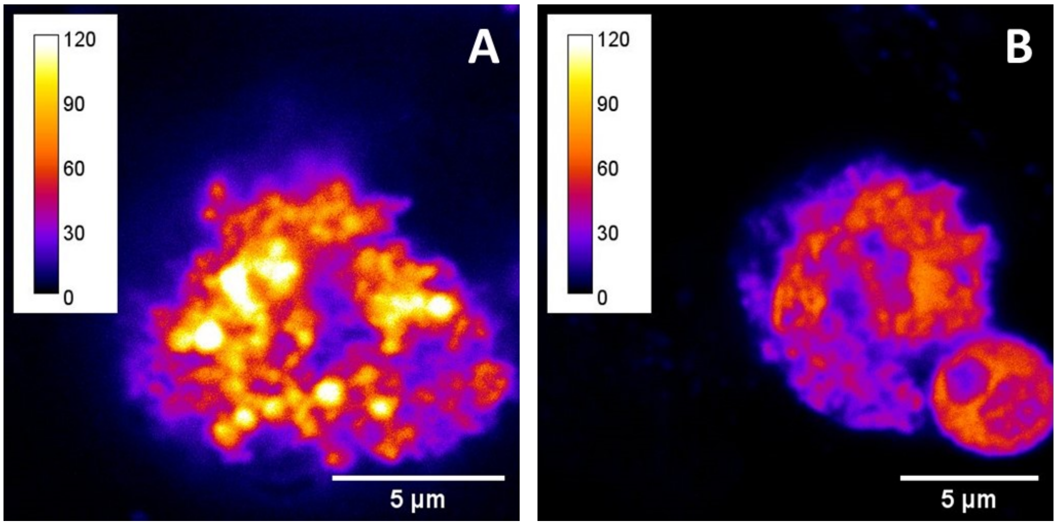

Figure S20. **(A)** and **(B)** depict confocal images of Ru-RHAU (25 µM, 24 h) in live **HeLa** cells, showing Ru-RHAU intensity throughout the SGs. These images indicate a similar intensity pattern as in the LSM airyscan super-resolution imaging shown in Figure 4 and were acquired using the Leica DMi8 confocal microscope.

Immunostaining

Coverslips were coated using poly-d-lysine for 30 min and allowed to dry completely. HeLa cells were seeded at 1×10^5 cells/mL on sterile, coated coverslips (round, Ø: 18 mm) in a 12-well tissue culture plate (Corning) and incubated for 24 h at 37 ^{o}C. Controls were fixed immediately (untreated controls) or incubated at 42 ^{o}C for 1 h to induce SGs prior to fixation and test cells were treated with Ru-RHAU at 40 µM for 6 h or 25 µM for 24 h before fixation.

Cells were fixed with 4% paraformaldehyde (BioLegend) for 15 minutes at room temperature (RT), and subsequently permeabilized with 0.01% Triton X-100 in PBS (Invitrogen) for 5 min at RT. Cells were washed with PBS and blocked with 5% BSA in PBS (5% goat serum in PBS for BG4 studies) for 1 h at RT in the dark. The cells were incubated with Alexa-Fluor 647 conjugated G3BP1 antibody, Alexa-Fluor 647 conjugated TIA-1 antibody (Santa Cruz Biotechnology, 1:50) or anti-G4 antibody BG4 (Merck, 1:50) overnight at 4 ^{o}C. Cells stained with TIA-1 & G3BP1 were washed twice with PBS and Hoechst 33342 was added for 7 minutes at RT to stain the nucleus. BG4 stained cells were incubated with Flag-Tag (Cell Signaling Technology, 1:500, anti-rabbit mAb) for 2 hours at RT, followed by a 3 h incubation with goat anti-rabbit AlexaFluor 647 (Invitrogen, 1:500) at RT, and Hoechst 33342 (2 µg/mL) for 7 minutes at RT. Cells were washed twice with PBS between each incubation. Coverslips were mounted on glass slides using Prolong Gold Antifade (Invitrogen), incubated for 24 h at RT in the dark and sealed prior to confocal imaging.

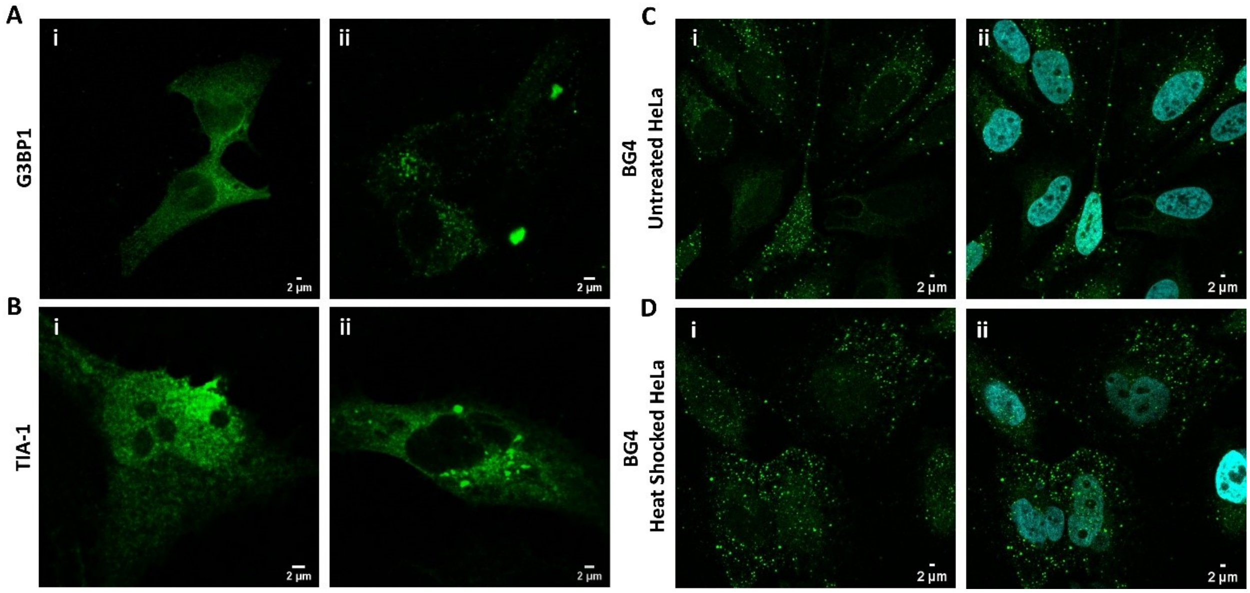

Figure S21. Immunostaining controls in fixed **HeLa** cells showing **(A)** G3BP1 and **(B)** TIA-1 in **(i)** untreated cells and **(ii)** cells treated by heat shock (42 °C, 1 h) prior to fixation and **(C)** untreated control cells with BG4 or cells treated by heat shock prior to fixation and BG4 staining (with **(i)** and without **(ii)** Hoechst 33342 nuclear staining).

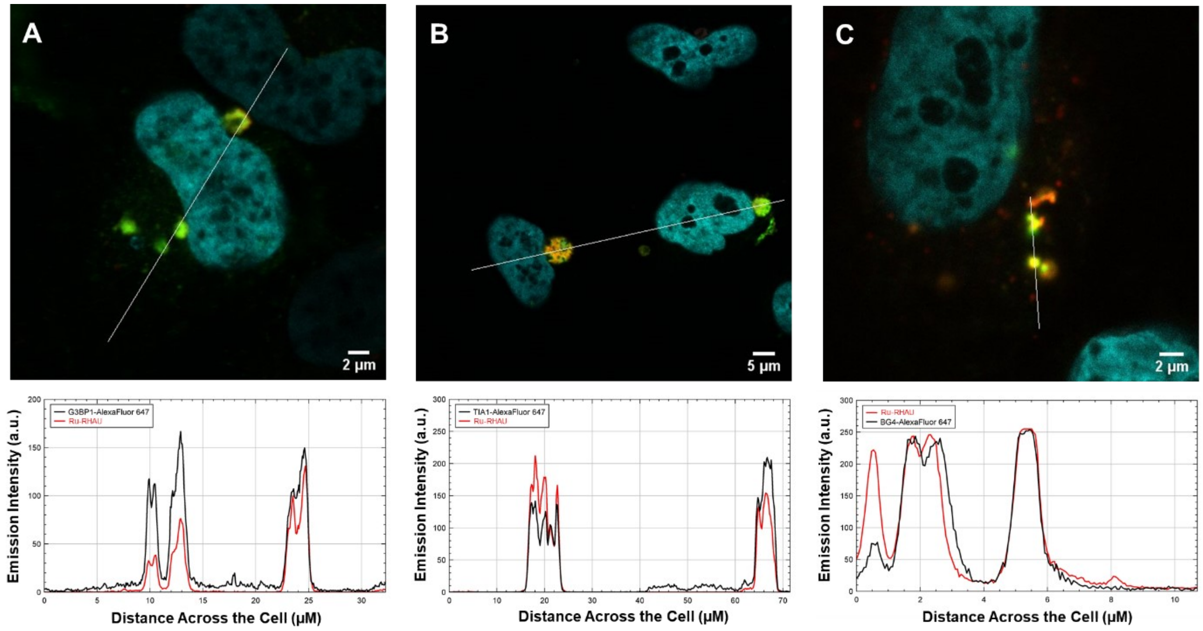

Figure S22. Co-localization of **HeLa** cells with Ru-RHAU (25 µM, 24 h) and **(A)** G3BP1, **(B)** TIA-1 or **(C)** BG4. The ROI used to generate the co-localization graphs is indicated by the white lines in each image.

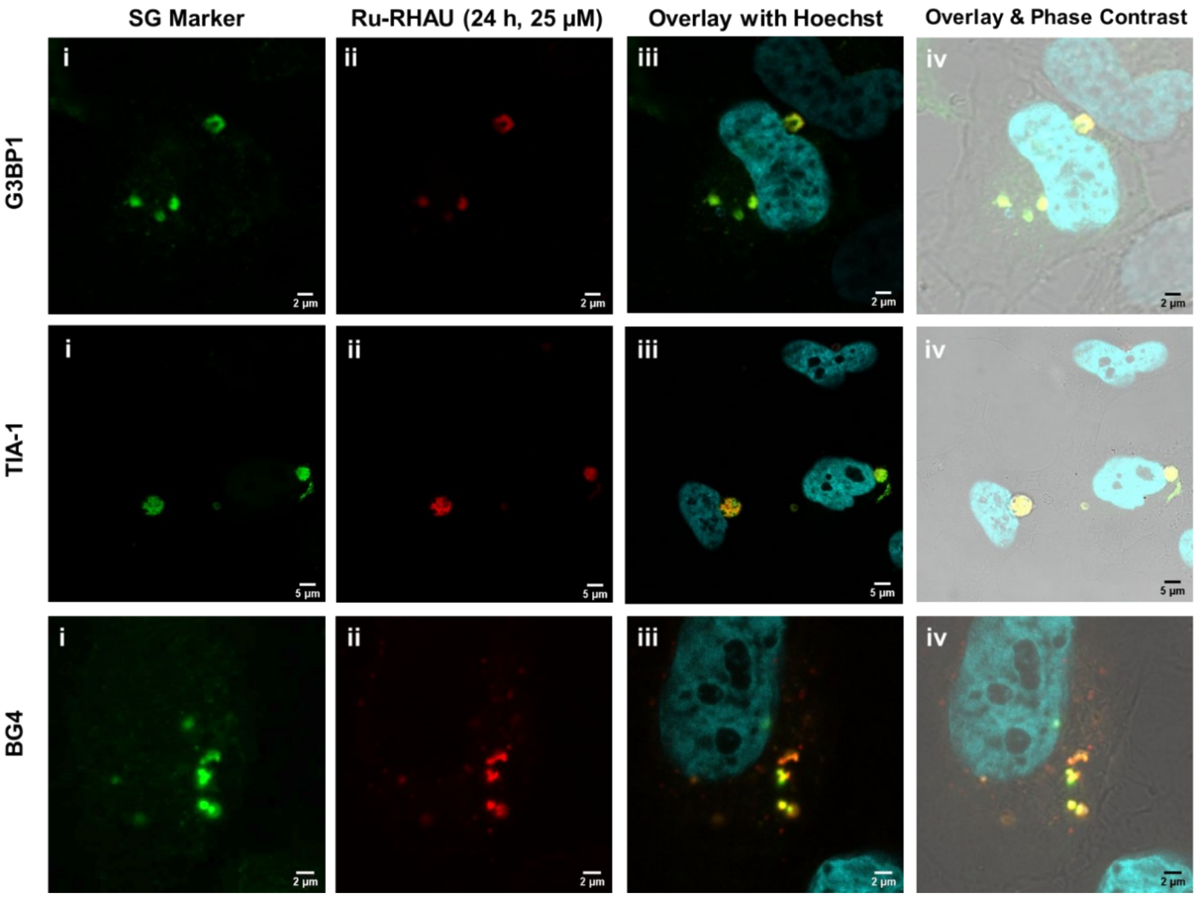

Figure S23. Confocal imaging of immunostaining of the individual channels in Figure S20, where **(i)** depicts antibody staining with AlexaFluor 647, **(ii)** shows Ru-RHAU staining and **(iii-iv)** is the overlay of **(i)** and **(ii)** with Hoechst 33342 nuclear stain (2 µg/mL, 7 min) and the corresponding phase contrast image. 100x oil.

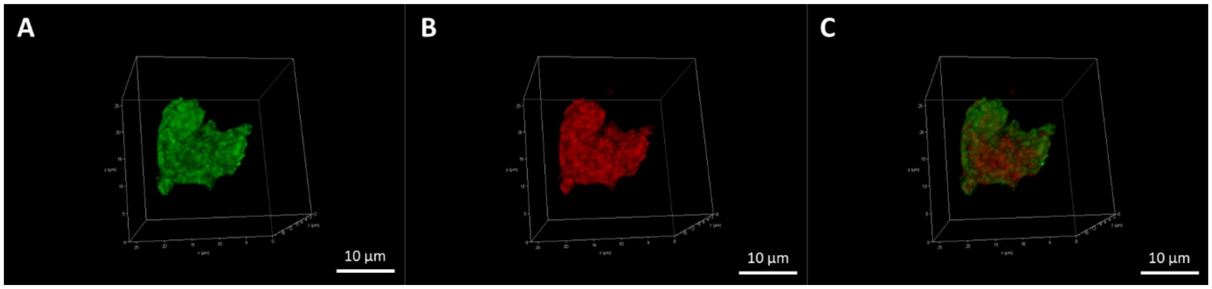

Figure S24. 3D rendering of a SG in a **HeLa** cell stained with **(A)** TIA-1 (1:50), **(B)** Ru-RHAU (25 µM, 24 h) and **(C)** the overlay. 100x oil.

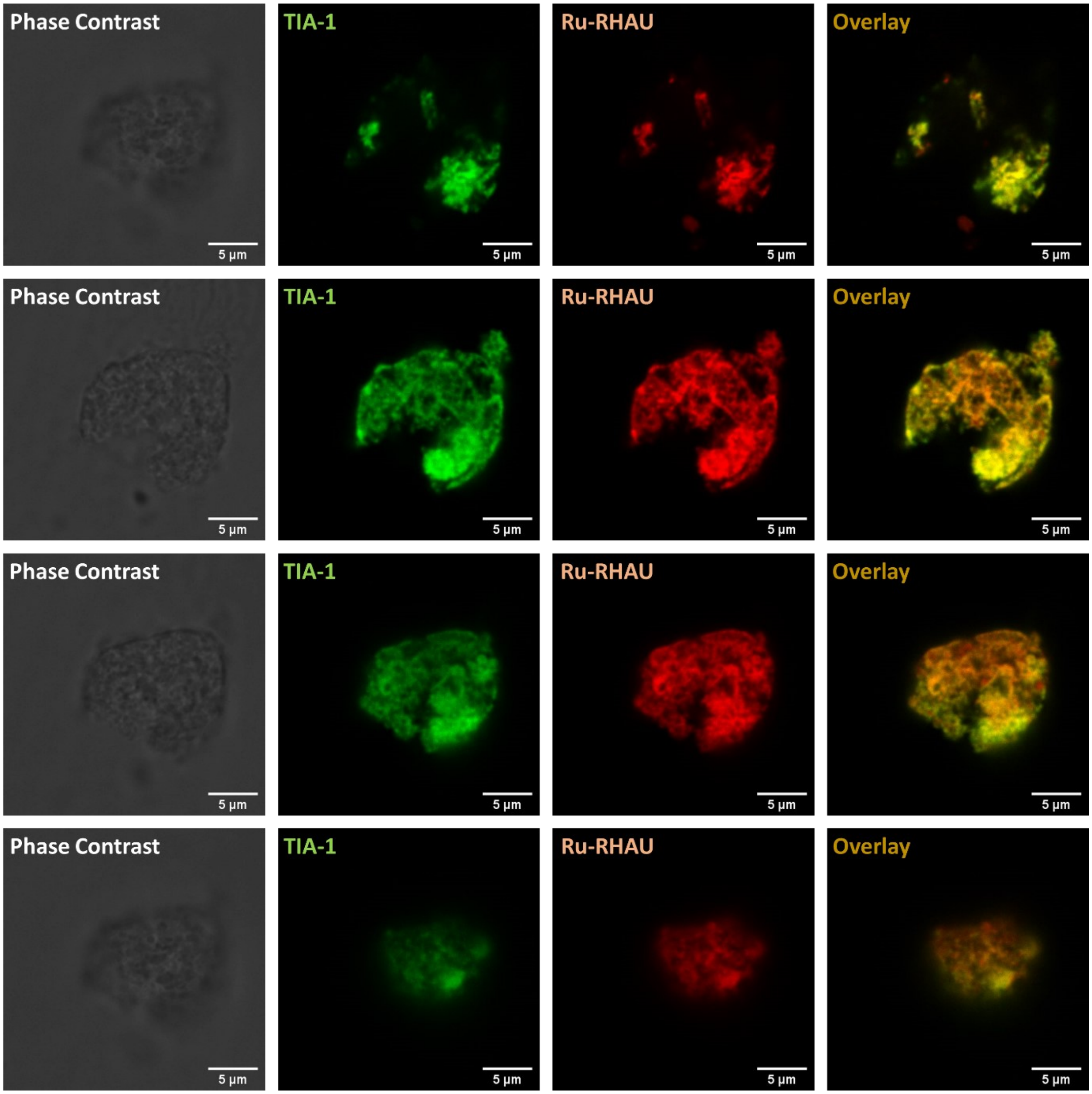

Figure S25. Confocal imaging of individual z's (TIA-1, Ru-RHAU, the overlay and corresponding phase contrast channel) used to create a 3D reconstruction image in Figure S23.

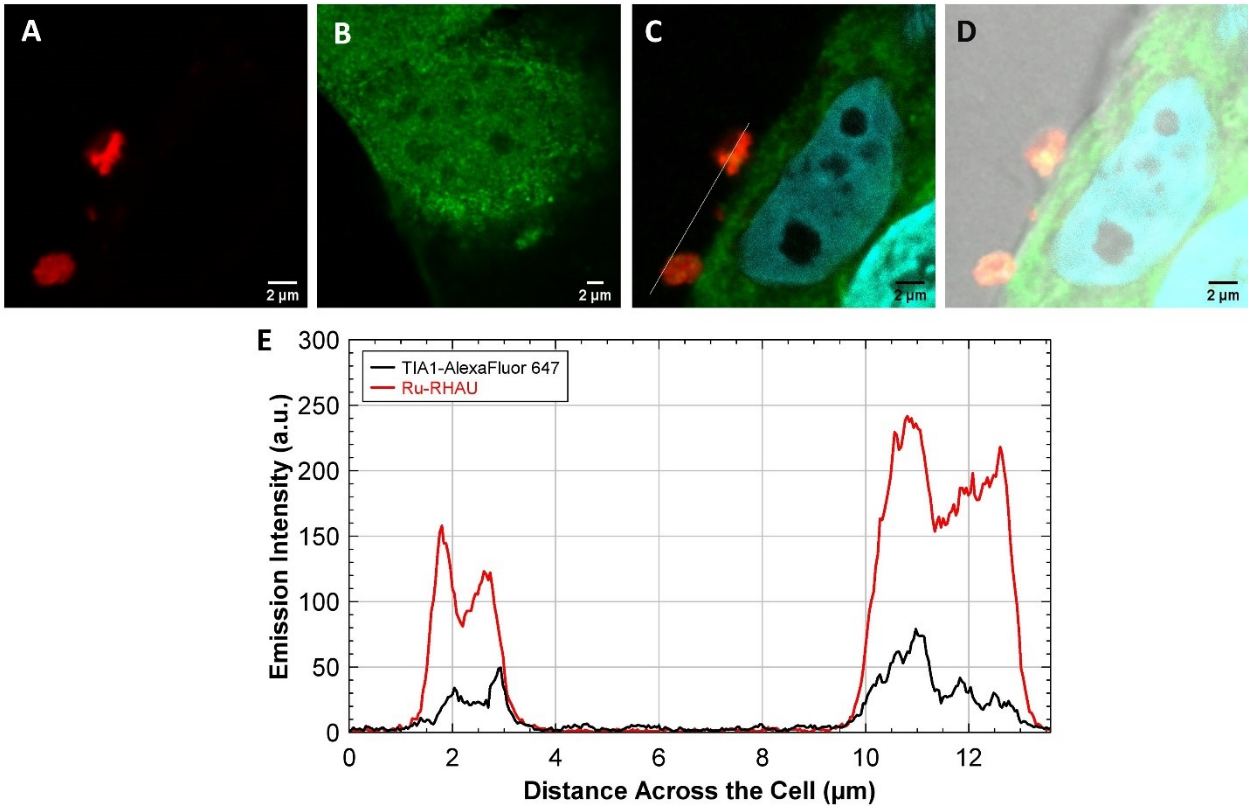

Figure S26. Confocal imaging of **MCF-7** cells treated with **(A)** Ru-RHAU (40 µM, 6 h) and immunostained with **(B)** TIA-1 where **(C)** is the overlay of the channels with Hoechst 33342 nuclear stain (2 µg/mL, 7 min) and **(D)** with the corresponding phase contrast image. The plot profile **(E)** was generated using the ROI indicated by the white line in (C). 100x oil.

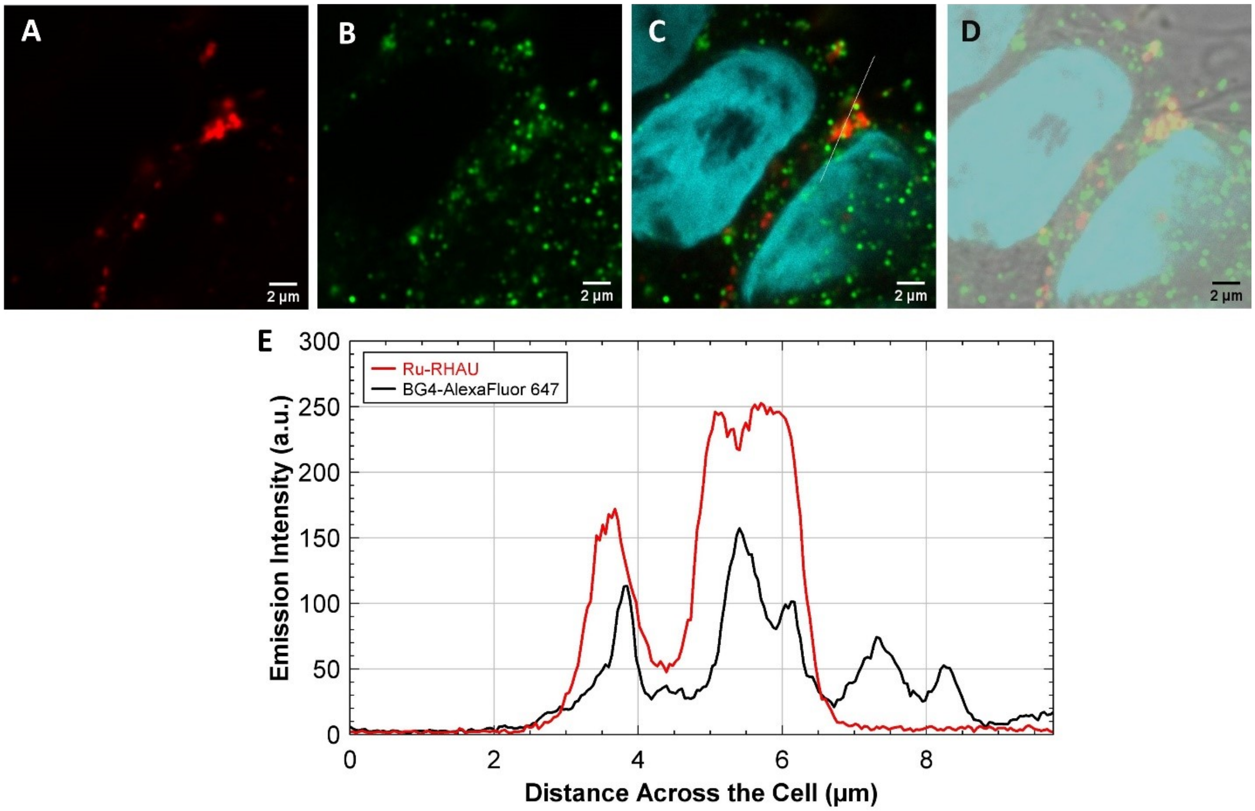

Figure S27. Confocal imaging of **MCF-7** cells treated with **(A)** Ru-RHAU (40 µM, 6 h) and immunostained with **(B)** BG4 where **(C)** is the overlay of the channels with Hoechst 33342 nuclear stain (2 µg/mL, 7 min) and **(D)** with the corresponding phase contrast image. The plot profile **(E)** was generated using the ROI indicated by the white line in (C). 100x oil.

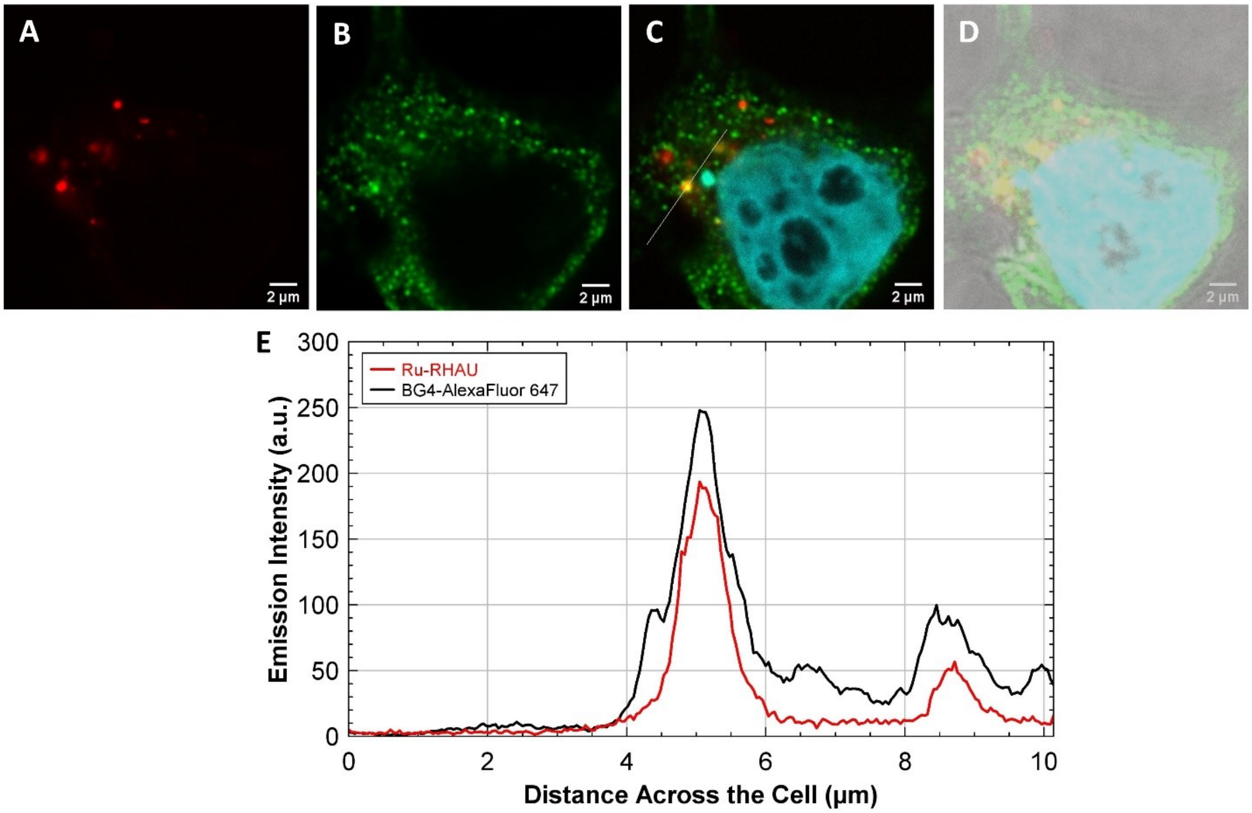

Figure S28. Confocal imaging of **MCF-7** cells treated with **(A)** Ru-RHAU (25 µM, 24 h) and immunostained with **(B)** BG4 where **(C)** is the overlay of the channels with Hoechst 33342 nuclear stain (2 µg/mL, 7 min) and **(D)** with the corresponding phase contrast image. The plot profile **(E)** was generated using the ROI indicated by the white line in (C). 100x oil.

SG Disassembly

HeLa cells were prepared as per routine imaging studies and incubated with either 40 µM (6 h) or 25 µM Ru-RHAU (24 h). Cells were imaged every 15 minutes for 2.5 h to monitor the disassembly of SGs over time. Hoechst 33342 was added to stain the nucleus prior to imaging. N=2.

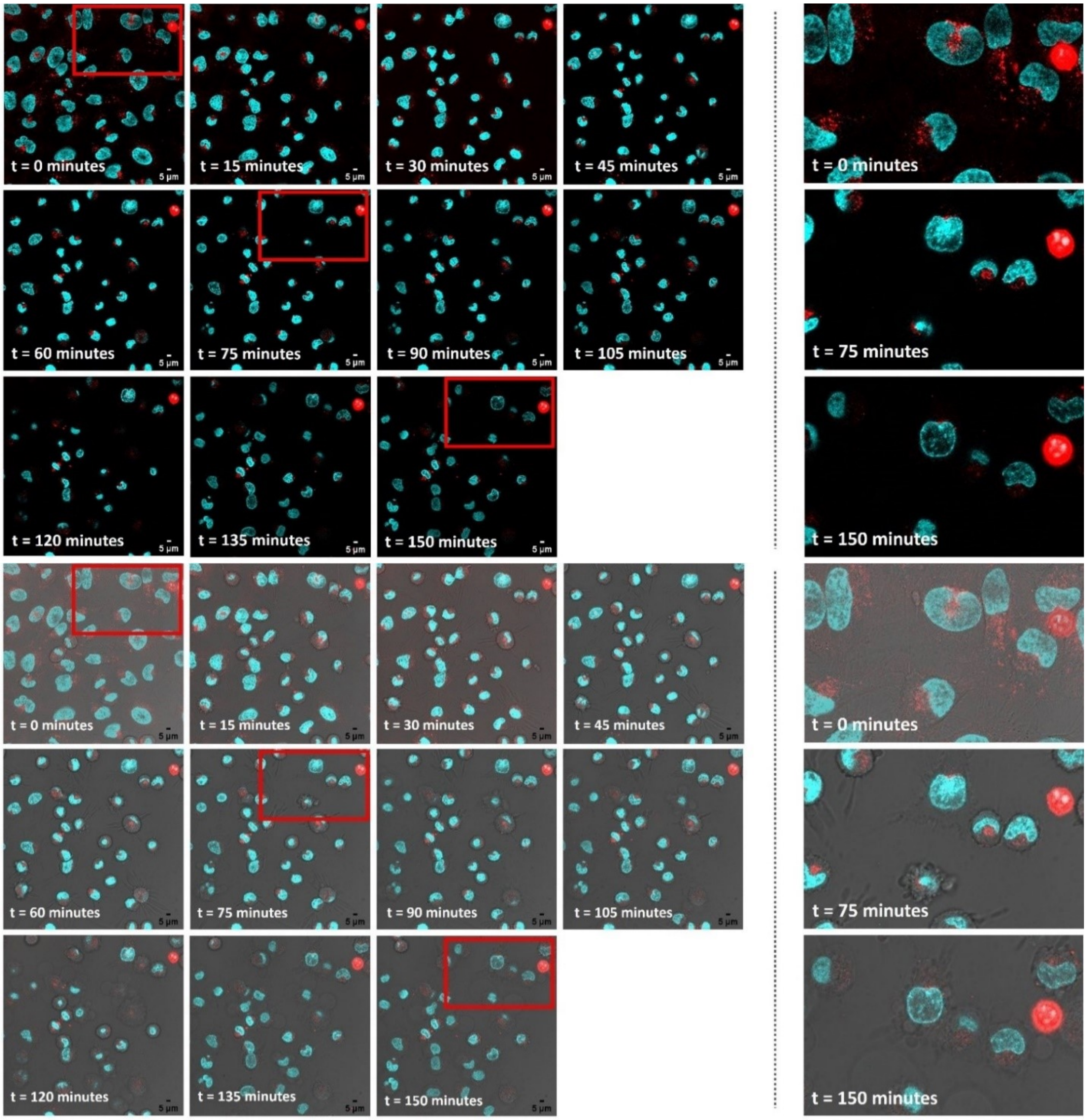

Figure S29. Disassembly over Ru-RHAU (40 μM, 6 h) in live HeLa cells over time with corresponding phase contrast images in gray, indicating changes in cell morphology over time. Timings are indicated on each image where t = the time since Ru-RHAU has been removed. Zoomed images at t = 0, 75 and 150 minutes are indicated by a red box and shown in the columns on the right. Scale bars read 5 μm.

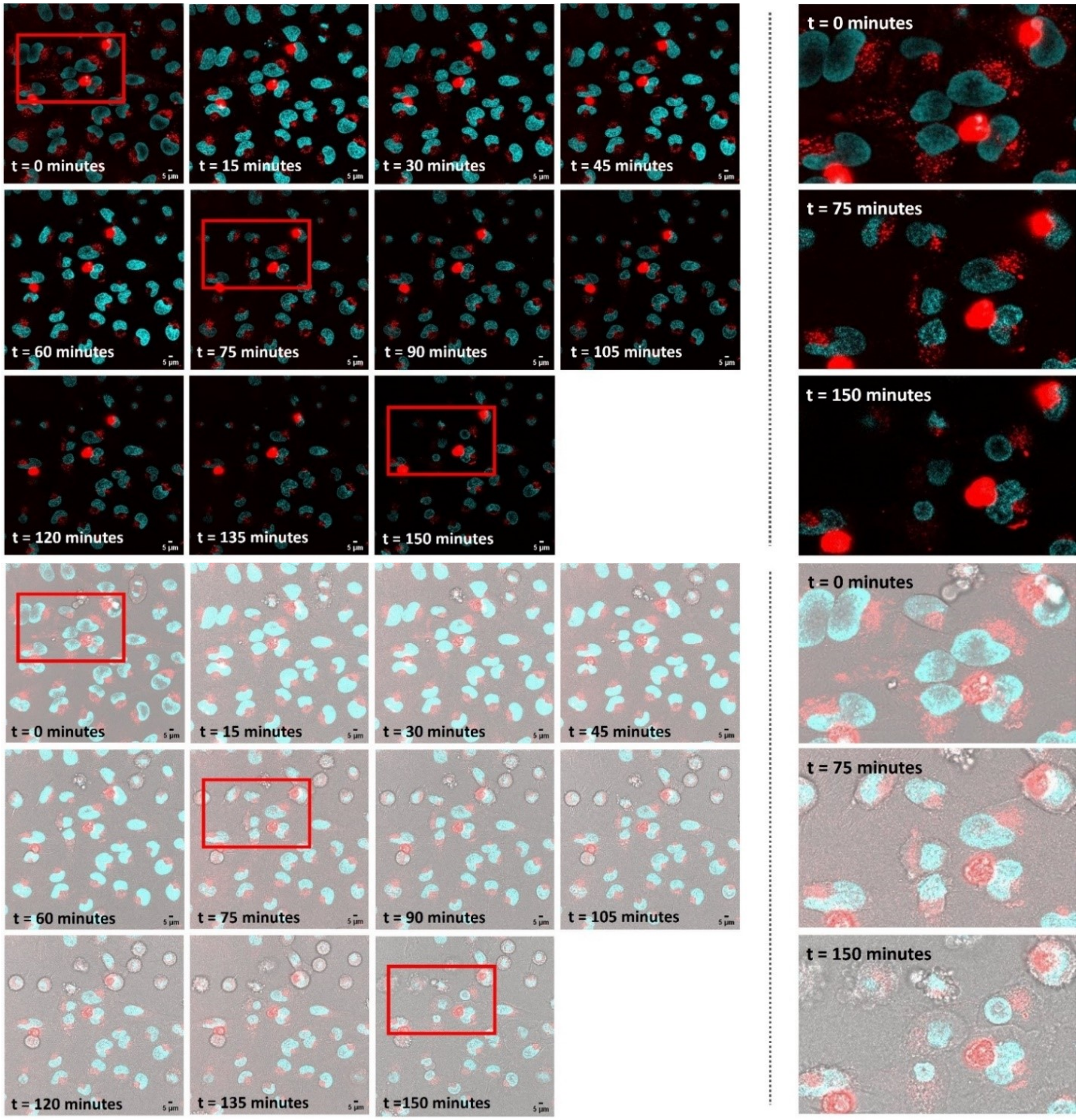

Figure S30. Disassembly of Ru-RHAU (25 μM, 24 h) in live HeLa cells over time with corresponding phase contrast images in gray, indicating changes in cell morphology over time. Timings are indicated on each image where t = the time since Ru-RHAU has been removed. Zoomed images at t = 0, 75 and 150 minutes are indicated by a red box and shown in the columns on the right. Scale bars read 5 μm.

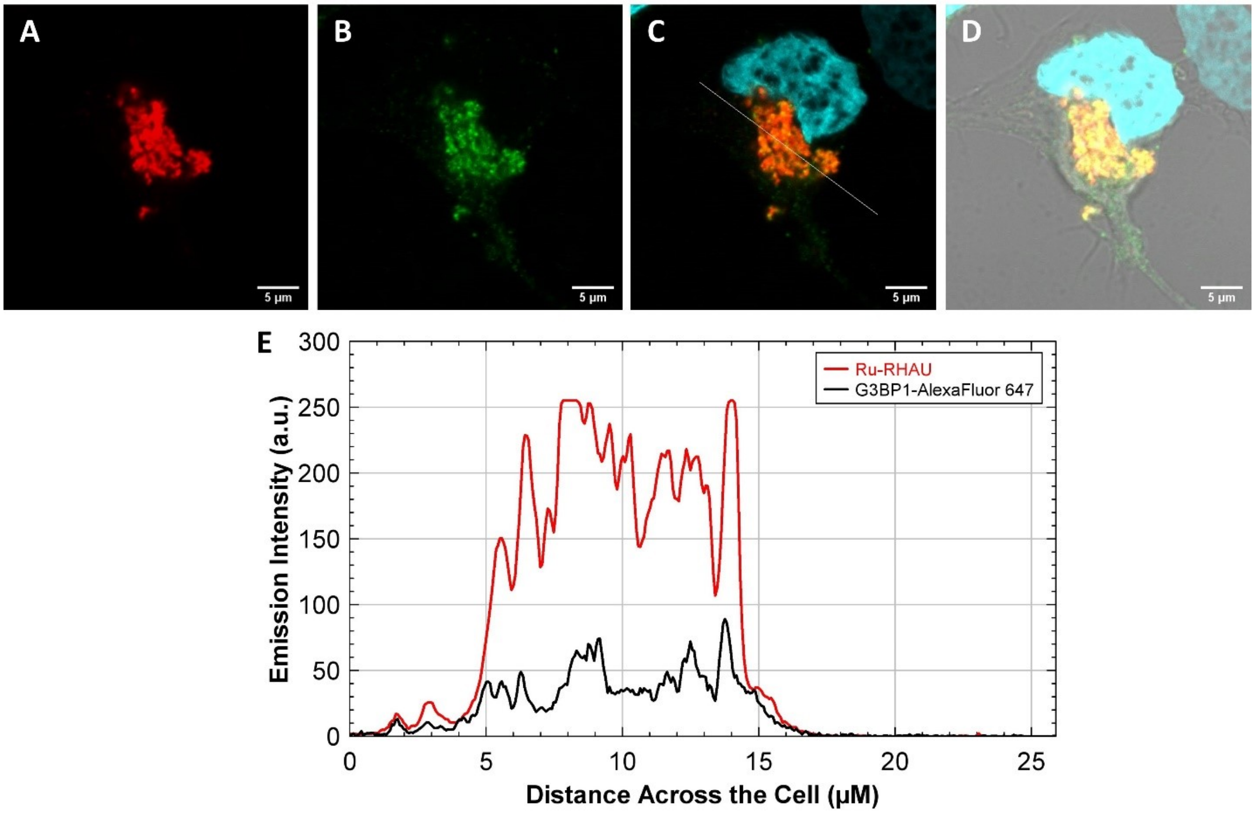

Figure S31. Large pathological SG in a fixed **HeLa** cell stained with **(A)** Ru-RHAU 25 µM, 24 h and **(B)** G3BP1 and the corresponding overlay with **(C)** Hoechst 33342 (2 µg/mL, 7 minutes) nuclear staining and **(D)** corresponding phase contrast channel. The ROI used to generate the co-localization graph in **(E)** is indicated by the white line in (C).

Ru-RHAU with Heat Shock Treatment

HeLa cells were seeded at 1×10^5 cells/mL in 8 chamber slides (Ibidi, Germany) and allowed to grow for 24 h at 37 °C with 5% CO_2. The cells were treated with heat shock (42 °C) for 1 h in phenol red free media to induce stress granules. The cells were imaged immediately after the addition of Ru-RHAU (10 µM) at a heated stage set to 42 °C. Ru-RHAU was excited at 488 nm and the emission range set to between 550 and 800 nm. DRAQ7 (3 µM) was added each well prior to imaging to confirm cell viability. MCF-7 cells were seeded at 1×10^5 cells/mL in 8 chamber slides and allowed to grow for 48 h at 37 °C with 5% CO_2 and treated with heat shock and Ru-RHAU using the same protocol.

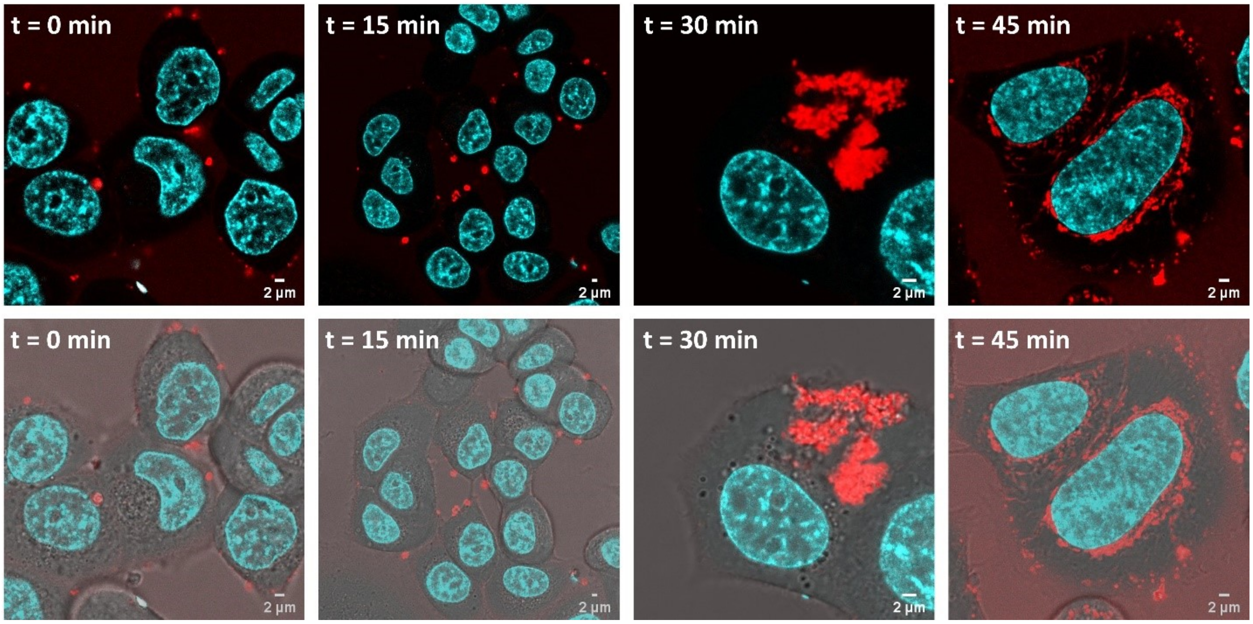

Figure S32. Confocal imaging of Ru-RHAU (10 µM) in **MCF-7** cells that were pre-treated by heat shock (42 °C, 1 h) at t = 0, 15, 30 and 45 minutes after the addition of Ru-RHAU and their respective overlays with the phase contrast channel. DRAQ7 (3 µM) was added to the cells and the lack of uptake confirmed good cell viability under imaging conditions.

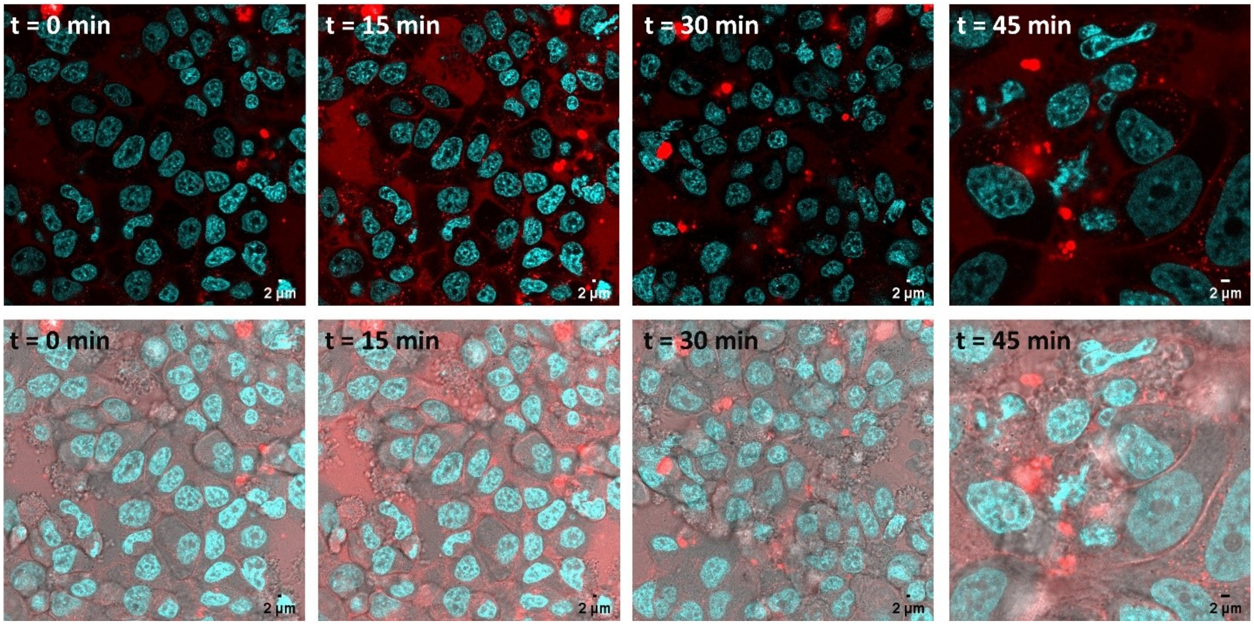

Figure S33. Confocal imaging of Ru-RHAU (10 µM) in **HeLa** cells that were pre-treated by heat shock (42 °C, 1 h) at t = 0, 15, 30 and 45 minutes after the addition of Ru-RHAU and their respective overlays with the phase contrast channel. Images after t = 0 and t = 30 minutes uptake are also shown in Figure 6. DRAQ7 (3 µM) was added to the cells and the lack of uptake confirmed good cell viability under imaging conditions.

Ru-RHAU with External Stress Stimuli

investigate the behavior of Ru-RHAU in the presence of stressors other than heat shock, a combination of glycolysis and mitochondrial inhibition were employed. 2-deoxy-d-glucose (2DG, 50 mM, 40 minutes) was used to inhibit glycolysis and either oligomycin (5 µM), sodium azide (NaN_3, 10 mM) or CCCP (carbonyl cyanide m-chlorophenyl hydrazone, 10 µM) added with the 2DG to inhibit mitochondria. The HeLa cells containing Ru-RHAU (25 µM, 24 h incubation) were washed twice with 1X PBS (37 °C) prior to treatment with the external stressors. The cells were imaged immediately after the 40-minute incubation with the external stressors. Similar large punctate foci were observed in cells treated with the Ru(II) complex alone, as with the addition of the extra stressors.

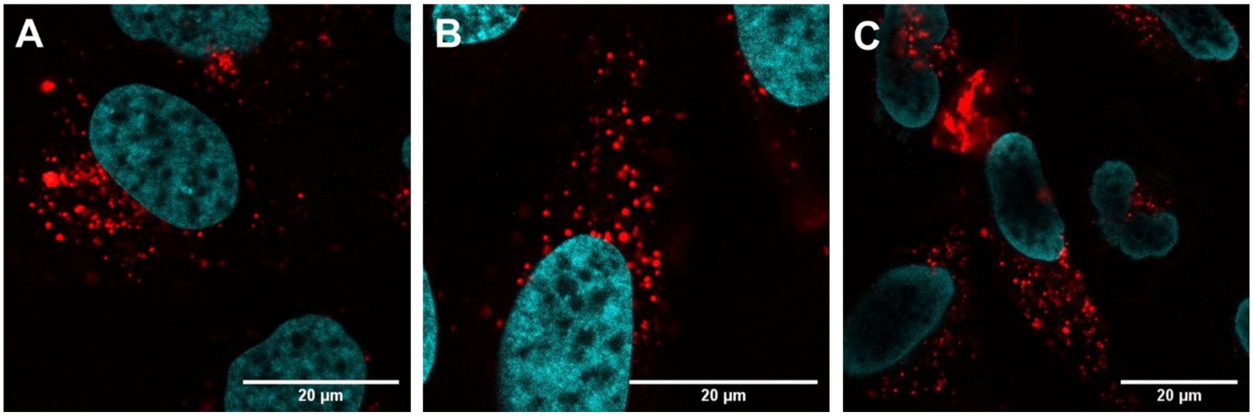

Figure S34. Live **HeLa** cells with Ru-RHAU (25 µM, 24 h) treated with 2DG in combination with **(A)** Sodium Azide, **(B)** CCCP and **(C)** Oligomycin for 40 minutes at 37 °C. Hoechst 33342 stains the nucleus.

Cytotoxicity Studies

HeLa cells were seeded in clear cell culture treated 96 well plates (Nunc) at 1 x 10^5 cells/mL and left to grow and attach for 24 h at 37 °C and 5 % CO_2. The cells were then treated with 100, 75, 60, 50, 25, 15, 10 and 5 µM Ru-RHAU. Six replicate wells were treated for each concentration, and the plates incubated for 24 h or 6 h in the dark at 37 °C, additionally cells were incubated for 24 or 6 h and a 2 h incubation added to enable SG disassembly before assessing viability. Cell viability of all plate-based studies was assessed using the MTT (3-(4,5-Dimethylthiazol-2-yl)-2,5-Diphenyltetrazolium Bromide) assay. The wells were emptied on the day of the viability assay (i.e. after 24 h Ru incubation) and replaced with 100 µL phenol red free media. 25 µL of a 2.5 mg/mL MTT solution (Invitrogen) was added per 100 µL of media and incubated at 37 °C for 3 h in the absence of light. During the 3 h incubation, actively respiring cells convert the yellow MTT into formazon, a water-insoluble purple crystal. The MTT/media solution was carefully removed from the wells and replaced with 100 µL DMSO to solubilize the formazon. Absorbance was measured using a BMG LABTECH CLARIOstar plate reader at 550 nm and 620 nm (corrected for background subtraction), with a 5-minute shaking step in the plate reader prior to any measurements. Cell viability is presented as a percentage (%) compared to untreated control cells. Plates were performed in duplicate.

Phototoxicity Studies

Phototoxicity plates were performed as described above. HeLa cells were treated with Ru-RHAU at 25, 22.5, 20, 17.5, 15, 10 and 5 μM for 24 h. The wells were then emptied and 100 μL phenol red free media was added. Cells were irradiated at a total dose of 5 ± 0.29 J/cm^2 (0.5 h at 2.63 ± 0.16 mW/cm^2) using a 470 nm LED (TeleOpto LEDA-X LED driver and array). The cells were incubated overnight after irradiation to facilitate cell recovery. The irradiation dose was established using a power meter (Edmund Optics, Coherent LaserCheck™). Cell viability was determined using the MTT assay.

Autophagy Assay

For plate-based autophagy studies HeLa cells were seeded in black cell culture treated 96 well plates (Nunc) at 2 x 10^5 cells/mL and allowed to grow and attach for 24 h at 37 °C and 5 % CO_2. For cytotoxicity studies, the cells were then treated with 15, 25, 50 or 60 μM Ru-RHAU, and the plates incubated for 24 h in the dark at 37 °C. A solution of 500 nM Rapamycin and 10 μM Chloroquine (in phenol red free media supplemented with 5% FBS) was used as a positive control (cells treated for 17 h) and untreated cells in media were used as a negative control. Autophagy was assessed using an autophagy assay kit (abcam), which measures autophagic vacuoles. Cells were washed with 1X apoptosis buffer supplemented with 5% FBS before a 30-minute incubation with the dual detection reagent (1 μL/mL green detection reagent and 1 μL/mL Hoechst-33342). The cells were washed twice with 200 μL apoptosis buffer supplemented with 5% FBS, 100 μL of 1X apoptosis buffer added to each well and the plate read immediately. Fluorescence was measured using a BMG LABTECH CLARIOstar plate reader at Ex 480 nm / Em 530 nm for the green detection reagent and Ex 340 nm / Em 480 nm for Hoechst 33342. Autophagy is presented as a percentage (%) increase of green reagent intensity compared to untreated control cells, and cytotoxicity is presented as a % decrease of Hoechst-33342 compared to untreated control cells, where a decrease of ≥ 30% indicates cytotoxicity. All conditions were performed in triplicate.

FAM-FLICA Apoptosis Assay

The FAM-FLICA polycaspase assay (ImmunoChemistry Technologies) can detect a range of activated caspases, including caspase-1, -2, -3, -4, -5, -6, -7, -8 and -9. HeLa cells were seeded at 3×10^5 cells/mL in a black bottomed 96 well plate (Nunc) at a total volume of 100 μL per well and incubated overnight at 37 °C with 5% CO_2. A solution of Staurosporine (1 μM/3 h) in cell culture media was prepared as a positive control and untreated cells were used as a negative control. Experimental populations were exposed to the Ru-RHAU at 50 and 60 μM for 6 or 24 h in the dark. After the incubation with Ru-RHAU, both the test and control cell populations were incubated with the FLICA dye for 60 min at 37 °C and washed using the buffer provided as per manufacturer's protocol. All conditions were performed in triplicate and analyzed using a BMG Labtech ClarioSTAR plate reader at Ex 488 nm / Em 520 nm.

References

[1] B. P. Sullivan, D. J. Salmon, T. J. Meyer, *Inorg. Chem.* **1978**, *17*, 3334–3341.

[2] U. Neugebauer, Y. Pellegrin, M. Devocelle, R. J. Forster, W. Signac, N. Moran, T. E. Keyes, *Chem. Commun.* **2008**, 5307–5309.

[3] K. Adamson, C. Dolan, N. Moran, R. J. Forster, T. E. Keyes, *Bioconjug. Chem.* **2014**, *25*, 928–944.

[4] A. Marchand, V. Gabelica, *Nucleic Acids Res.* **2016**, *44*, 10999–11012.

[5] F. D'Aria, V. M. D'Amore, F. S. Di Leva, J. Amato, M. Caterino, P. Russomanno, S. Salerno, E. Barresi, M. De Leo, A. M. Marini, S. Taliani, F. Da Settimo, G. F. Salgado, L. Pompili, P. Zizza, S. Shirasawa, E. Novellino, A. Biroccio, L. Marinelli, C. Giancola, *Eur. J. Pharm. Sci.* **2020**, *149*, 105337.

[6] R. V. Reshetnikov, J. Sponer, O. I. Rassokhina, A. M. Kopylov, P. O. Tsvetkov, A. A. Makarov, A. V. Golovin, *Nucleic Acids Res.* **2011**, *39*, 9789–9802.

[7] K. McQuaid, J. P. Hall, L. Baumgaertner, D. J. Cardin, C. J. Cardin, *Chem. Commun.* **2019**, *55*, 9116–9119.

[8] I. Renard, M. Grandmougin, A. Roux, S. Y. Yang, P. Lejault, M. Pirrotta, J. M. Y. Wong, D. Monchaud, *Nucleic Acids Res.* **2019**, *47*, 5502–5510.
